# Uncovering causal relationships in single-cell omic studies with causarray

**DOI:** 10.1093/bib/bbag175

**Published:** 2026-04-15

**Authors:** Jin-Hong Du, Maya Shen, Hansruedi Mathys, Kathryn Roeder

**Affiliations:** Department of Statistics and Actuarial Science, The University of Hong Kong, Pok Fu Lam, Hong Kong SAR 00000, China; Musketeers Foundation Institute of Data Science, The University of Hong Kong, Pok Fu Lam, Hong Kong SAR 00000, China; Department of Statistics and Data Science, Carnegie Mellon University, 5000 Forbes Ave, Pittsburgh, PA 15213, United States; Department of Neurobiology, University of Pittsburgh, 4200 Fifth Ave, Pittsburgh, PA 15261, United States; Department of Statistics and Data Science, Carnegie Mellon University, 5000 Forbes Ave, Pittsburgh, PA 15213, United States; Computational Biology Department, Carnegie Mellon University, 5000 Forbes Ave, Pittsburgh, PA 15213, United States

**Keywords:** causal inference, confounder adjustment, counterfactual, differential expression analysis, semiparametric inference

## Abstract

Advances in single-cell sequencing and Clustered Regularly Interspaced Short Palindromic Repeats (CRISPR) technologies have enabled detailed case-control comparisons and experimental perturbations at single-cell resolution. However, uncovering causal relationships in observational genomic data remains challenging due to selection bias and inadequate adjustment for unmeasured confounders, particularly in heterogeneous datasets. To address these challenges, we introduce causarray, a robust causal inference framework for analyzing array-based genomic data at both pseudo-bulk and single-cell levels under unmeasured confounding. causarray integrates a generalized confounder adjustment method to account for unmeasured confounders and employs semiparametric inference with flexible machine learning techniques to ensure robust statistical estimation of treatment effects. Benchmarking results show that causarray robustly separates treatment effects from confounders while preserving biological signals across diverse settings. We also apply causarray to two single-cell genomic studies: (i) an *in vivo* Perturb-seq study of autism risk genes in developing mouse brains and (ii) a case-control study of Alzheimer’s disease (AD) using three human brain transcriptomic datasets. In these applications, causarray identifies clustered causal effects of multiple autism risk genes and consistent causally affected genes across AD datasets, uncovering biologically relevant pathways directly linked to neuronal development and synaptic functions that are critical for understanding disease pathology.

## Introduction

The advent of genomic research has revolutionized our understanding of biological systems and disease mechanisms. In particular, advances in single-cell RNA sequencing (scRNA-seq) have provided unprecedented resolution of gene expression at the cellular level, enabling detailed characterization of cellular heterogeneity and its relevance to health and disease [1–4]. Likewise , understanding the regulatory circuits that govern the phenotypic landscape of human cells has long been considered a formidable challenge, but recent experimental innovations, such as pooled CRISPR-based perturbation assays, are making this goal increasingly attainable [5].

Fully realizing the potential of these technologies, however, requires analytical frameworks that move beyond mere association to uncover causal relationships at single-cell resolution [6–8]. Association studies identify correlations between treatments and outcomes, whereas causal inference seeks to estimate the effect of an intervention on an outcome. A widely used approach for causal inference is the potential outcomes framework, which contrasts observed outcomes with their unobserved counterparts, the counterfactuals, to quantify causal effects [8, 9]. Developing such causal models is essential for understanding biological processes and disease mechanisms, with important implications for treatments, precision medicine, genomic medicine, and related fields [10, 11].

One of the primary challenges in leveraging scRNA-seq and CRISPR data for causal inference is the presence of unmeasured confounders due to biological factors, such as correlated gene expression, and technical factors, such as batch effects. Furthermore, most genomic studies are observational in nature. Unlike randomized controlled trials, observational studies lack complete knowledge of the disease or treatment assignment mechanism, leading to potential biases in counterfactual estimation. In CRISPR screens [5, 12–14], perturbed cells are contrasted with non-targeting controls, but assignment is not fully random: continuous, cell-level variables such as cell size or differential exposure can leak into effect estimates, a problem amplified *in vivo* where more complex cellular environments exacerbate confounding. Although *in vivo* CRISPR screens are feasible [15, 16], many screens still rely on a few well-studied cell lines [17], limiting generalizability. As perturbation technologies scale, including multimodal assays [5], the need for robust causal inference that explicitly models unmeasured confounders becomes even more acute.

The presence of unmeasured confounders can undermine the validity of causal conclusions in observational studies [18, 19]. Existing methods for causal estimation, such as CoCoA-diff [7] and CINEMA-OT [20], rely on matching techniques that assume the causal structure is transferable between treatment and control groups. However, this assumption breaks down when covariate distributions differ significantly across groups, leading to biased estimates. On the other hand, to adjust for confounding and unwanted variation in statistical inference, other methods like surrogate variable analysis (SVA) [21] and remove unwanted variation (RUV) [22] assume additive relationships between covariates and outcomes via linear models. While effective for certain bulk RNA-seq datasets, these approaches often fail to capture the sparsity, zero inflation, and overdispersion inherent in single-cell genomic data [18, 23]. Tackling these challenges requires integrating robust confounder adjustment with flexible modeling techniques to ensure valid causal inference in complex genomic data.

**Figure 1:**
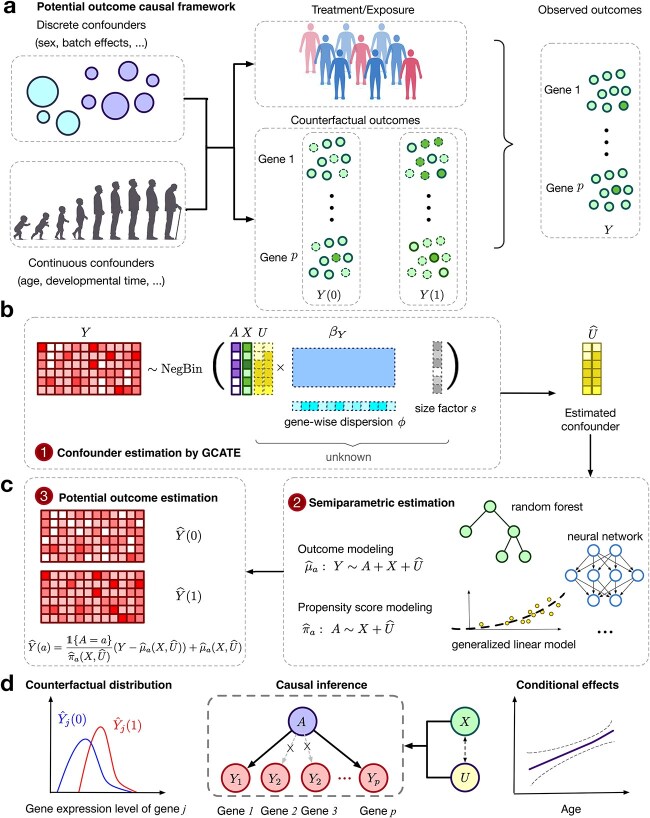
**Overview of the proposed causarray method.** (a) Illustration of the data generation process for pseudo-bulk and single-cell data. (b) In the first step, the gene expression matrix, $Y$, is linked to the treatment, $A$, measured covariates, $X$, and confounding variables, $U$, via a GLM model. The cell-wise size factor, $s$, and gene-wise dispersion parameter, $\phi $, are estimated from the data, and the unmeasured confounder $U$ is estimated by $\hat{U}$ through the augmented GCATE method. (c) In the second step, GLMs and flexible machine learning methods including random forest and neural network can be applied for outcome modeling ($\mathbb{E}[Y\mid A=a,W] = {\hat{\mu }}_{a}(W)$) and propensity modeling ($\mathbb{P}(A=a\mid W)=\hat{\pi }_{a}(W)$) using $W=[X,\hat{U}]$. In the third step, the predicted values of the estimated outcome and propensity score functions give rise to the estimated potential outcomes for each cell and each gene. (d) Downstream analysis includes contrasting the estimated counterfactual distributions, performing causal inference, and estimating the CATEs.

In response to these challenges, we introduce a new framework for applying causal inference in omic studies. Our approach leverages generalized factor models tailored to count data to account for unmeasured confounders, ensuring robust adjustment for unmeasured confounders while preserving biological signals. It further relies on the potential outcomes framework and employs a robust estimation procedure, which combines outcome and propensity score models to ensure reliable statistical inference even if one model is misspecified [24, 25]. This framework effectively addresses biases introduced by both observed and unobserved confounders, making it particularly well-suited for analyzing complex genomic data at both pseudo-bulk and single-cell levels (Fig. 1a). By integrating advanced statistical and machine learning techniques with a causal inference framework, our method enables a range of downstream analyses, including accurate estimation of counterfactual distributions, causal gene detection, and conditional treatment effect analysis. This approach not only improves the interpretability and precision of genomic analyses but also uncovers critical insights into gene expression dynamics under disease or perturbation conditions, advancing our understanding of underlying biological mechanisms.

We demonstrate the effectiveness of causarray through benchmarking on several simulated datasets, comparing its performance with existing single-cell-level perturbation analysis methods and pseudo-bulk-level differential expression (DE) analysis methods. Next, we apply causarray to two single-cell genomic studies: a Perturb-seq study investigating autism spectrum disorder/neurodevelopmental delay (ASD/ND) genes in developing mouse brains and a case-control study of Alzheimer’s disease (AD) using human brain transcriptomic datasets. For the AD analysis, we validate our findings across three independent datasets, showcasing the robustness and reproducibility of causarray in identifying causally affected genes and uncovering biologically meaningful pathways. These applications highlight the potential of causarray to advance our understanding of complex disease mechanisms through rigorous causal inference on general omics.

## Materials and methods

### Counterfactual imputation and inference

Our objective is to determine whether a gene is causally affected by a “treatment” variable after controlling for other technical and biological covariates, which may affect the treatment and outcome variables. Here, we use the term treatment generally; in the narrow sense, it can mean genetic and/or chemical perturbations [26, 27], such as CRISPR-CAS9, and, more broadly, it can mean the phenotype of a disease [7]. We acknowledge that while many differentially expressed genes can be considered a result of disease status, for most late-onset disorders, a smaller fraction of genes could have initiated disease phenotypes. Our method aims to determine the direct effects of treatments on modulated gene expression outcomes.

In observational data, the response variable can be confounded by measured and unmeasured biological and technical covariates, making it difficult to separate the treatment effect from other unknown covariates. As a consequence, it is challenging to draw causal inferences; even tests of association may lead to an excess of false discoveries and/or low power. Fortunately, the potential outcomes framework [24, 25] formulates general causal problems in a way that allows for the treatment effect to be separated from the effects of other variables. However, even this framework is challenged by unmeasured covariates. Before introducing our method for estimating unmeasured confounders, we first outline the general potential outcomes framework.

Consider a study in which $Y$ is the response variable and $A$ is the binary treatment variable for an observation. In the potential outcomes framework, $Y(a)$ is the outcome that we would have observed if we set the treatment to $A=a$. Naturally, we can only observe one of the two potential outcomes for each observation, so


\begin{equation*} Y = \mathbb{1}\{A = 1\}Y(1) + \mathbb{1}\{A = 0\}Y(0), \end{equation*}

In the context of a case-control study of a disease, this would answer the question: What is the expected difference in gene expression if an individual had the disease (case, $A=1$) versus if they did not (control, $A=0$)?

Semiparametric methods provide a powerful tool for estimating potential outcomes in observational studies where randomization is not possible [24, 25]. Specifically, we estimate two key quantities: (1) $\mu _{a}(X)$, the mean response of the outcome variable conditional on treatment $A=a$ and covariates $X=x$, and (2) $\pi _{a}(X)$, the propensity score, which is defined as the probability of receiving treatment $A=a$ given covariates $X$, i.e. $\pi _{a}(X) = \mathbb{P}(A=a\mid X)$. Using these estimates, we compute potential outcomes using the augmented inverse probability weighted estimator


\begin{align*} & \hat Y(a) = \frac{\mathbb{1}\{A=a\}}{\hat\pi_a(X)}(Y - \hat\mu_a(X)) + \hat\mu_a(X). \end{align*}

which provides a consistent estimate as long as *either* the outcome model, $\mu _{a}(X)$, or the propensity score model, $\pi _{a}(X)$, is correctly specified. Given this estimate, we can easily perform downstream inference tasks such as computing log fold change (LFC), and testing for causal effects on gene expressions (Fig. 1a). An advantage of this approach is that counterfactual imputation denoises/balances gene expression under two different conditions. Additionally, having access to estimated potential outcomes facilitates downstream analyses such as estimating causal effects conditional on measured confounders like age.

### The probabilistic modeling of confounders

A key step in these types of analyses is estimating unmeasured confounders. To adjust for confounding, factor models were popularized in surrogate variable analysis literature and have since been widely adopted in bulk gene expression studies [21]. We propose an improved version of generalized latent models [18] to identify potential unmeasured confounders, which extends traditional confounder adjustment methods by incorporating more flexible nonlinear models that better capture the unique characteristics of genomic count data, such as zero-inflation (an excess of zero counts) and over-dispersion (greater variability than expected under standard Poisson assumptions). These enhancements allow for more accurate modeling of gene expression data, addressing limitations of simpler linear models in high-dimensional genomic analyses. Using this generalized factor analysis approach, we estimate unmeasured confounders $U$ alongside potential outcomes (Fig. 1b and c), enabling direct estimation of downstream quantities such as LFC (Fig. 1d).

We restrict our modeling to one cell type at a time. Although the framework is, in principle, extensible to a joint analysis of all cell types, two practical considerations favor a per-type pipeline: (i) Computational burden. Pooling millions of cells across types inflates both the parameter space and the memory usage, making hyperparameter tuning prohibitively slow for large-scale screens. (ii) Dominance of marker-gene signal. When latent factors are learned on the pooled data, the strongest axes of variation are inevitably the marker genes that separate coarse cell identities. As a result, the leading factors reflect cell-type structure rather than the more subtle technical and treatment-related confounders we aim to remove. Preventing this would require *adhoc* marker filtering or explicit constraints, which can be error-prone.

Binning cells by type before estimating confounders, therefore, yields factors that capture within-type heterogeneity (batch, cell-cycle stage, library size, etc.) while leaving biologically meaningful differences between types intact. Whether one ultimately wishes to regress out cell-type variation depends on the scientific objective, but analysing each lineage separately provides a computationally tractable and statistically robust default.

For the $i$th observation (e.g. a single cell or sample) and the $j$th gene, we model the adjusted expression $\mu _{ij} = Y_{ij}/s_{i}$, where $Y_{ij}$ is the observed expression level, and $s_{i}$ is the size factor for the $i$th cell. The size factor accounts for differences in sequencing depth or library size across samples, ensuring that comparisons are not biased by technical variability. We assume that $\mu _{ij}$ follows an exponential family distribution, which is a flexible class of probability distributions commonly used in generalized linear models (GLMs) with density of $\mu _{ij}$ given by:


\begin{align*} & p(\mu_{ij}\mid \theta_{ij}) = h(\mu_{ij})\exp\left(\mu_{ij} \theta_{ij} - A(\theta_{ij})\right), \end{align*}

where $\theta _{ij}$ is the natural parameter that determines the mean and variance of $\mu _{ij}$, $h(\mu _{ij})$ is a known base measure, and $A(\theta _{ij})$ is the log-partition function, which ensures that the density integrates to 1. For negative binomial distributions, both the base measure $h(\theta _{ij}) = \choose{Y_{ij}+\phi _{j}-1}{Y_{ij}}$ and the log-partition function $A(\theta _{ij})= -\phi _{j}\log (1-e^{\theta _{ij}})$ depend on the dispersion parameter $\phi _{j}$ of the $j$th gene. The GLMs do not imply that the underlying biological or technical confounders act linearly on expression $Y_{ij}$. When the dependence between $(A,X)$ and the latent confounding is nonlinear, this working model can be viewed as estimating the best low-rank linear projection of that nonlinear nuisance variation in expression space, i.e. the component of unmeasured variation that most strongly explains residual correlation in $Y$ after accounting for $(A,X)$ and is therefore most relevant for bias reduction.

In matrix form, we model the natural parameters $\boldsymbol{\Theta } = (\theta _{ij}$  $)_{1\leq i\leq n, 1\leq j\leq p}$, as a decomposition into two components: $ \boldsymbol{\Theta } = \tilde{\boldsymbol{X}}\boldsymbol{B}^{\top } + \boldsymbol{U}\boldsymbol{\Gamma }^{\top }$. Here, $\tilde{\boldsymbol{X}} = [\boldsymbol{X}, \boldsymbol{A}] \in \mathbb{R}^{n\times (d+1)}$ combines observed covariates $\boldsymbol{X}$ (e.g. biological or technical factors) with treatment indicators $\boldsymbol{A}$, where $n$ is the number of observations, and $d$ is the dimension of $\boldsymbol{X}$; $\boldsymbol{B} \in \mathbb{R}^{p\times (d+1)}$ represents unknown regression coefficients for the effects of covariates and treatments on gene expression; $\boldsymbol{U} \in \mathbb{R}^{n\times r}$ represents latent variables capturing unmeasured confounders, where $r$ is the number of latent factors; and $\boldsymbol{\Gamma } \in \mathbb{R}^{p\times r}$ represents unknown coefficients linking unmeasured confounders to gene expression. This decomposition assumes that gene expression levels are influenced by both observed covariates ($\tilde{\boldsymbol{X}}$) and unmeasured confounders ($\boldsymbol{U}$). The term $\tilde{\boldsymbol{X}}\boldsymbol{B}^{\top }$ captures the effects of observed covariates and treatments, while $\boldsymbol{U}\boldsymbol{\Gamma }^{\top }$ captures the effects of unmeasured confounders. To estimate the dimension of unmeasured confounders $r$ and the corresponding unknown quantities ($\boldsymbol{B}$, $\boldsymbol{U}$, $\boldsymbol{\Gamma }$), we employ methods detailed in Supplementary Information S1.3. This includes techniques for estimating latent factors ($\hat{\boldsymbol{U}}$) and extending the framework to handle multiple treatments. Once these quantities are estimated, we treat $\boldsymbol{W} = [\boldsymbol{X}, \hat{\boldsymbol{U}}] \in \mathbb{R}^{d+r}$ as the complete set of confounding covariates—combining both observed covariates ($\boldsymbol{X}$) and estimated unmeasured confounders ($\hat{\boldsymbol{U}}$).

Single-cell transcriptomic data exhibit systematic variation from both technical and biological sources, even when samples are processed under a nominal “single batch.” Technical effects include library size and capture efficiency (addressed in part via size factors), as well as latent run/lane effects, ambient RNA, and QC-related artifacts. Biological heterogeneity within a cell type (e.g. cell-cycle state, activation/stress programs, and developmental state) also contributes substantial structured variation. Either source can act as a confounder when it influences both the treatment/exposure assignment $\boldsymbol{A}$ (e.g. disease status or perturbation condition) and gene expression outcomes $\boldsymbol{Y}$. In causarray, measured technical/biological variables can be included in $\boldsymbol{X}$ when available, while the remaining shared structure is captured by the estimated latent factors $\widehat{\boldsymbol{U}}$ under the generalized factor model $\boldsymbol{\Theta }=\widetilde{\boldsymbol{X}} \boldsymbol{B}^\top +\boldsymbol{U}\boldsymbol{\Gamma }^\top $, yielding $\boldsymbol{W}=[\boldsymbol{X},\widehat{\boldsymbol{U}}]$ for downstream semiparametric causal estimation.

### Semiparametric estimation

Throughout the paper, we consider the LFC as the target estimand:


\begin{align*} & \tau_j:= \log(\mathbb{E}[Y_j(1)]/\mathbb{E}[Y_j(0)]), \end{align*}

which quantifies the relative change in expected gene expression levels between treatment ($A=1$) and control ($A=0$) conditions for gene $j$. Extensions to other estimands are provided in Supplementary Information S2.2. The semiparametric estimation framework is a widely used approach that is agnostic to the underlying data-generating process. It provides valid estimation and inference results as long as either the conditional mean model ($\mu _{j}$) or the propensity score model ($\pi $) is correctly specified. This robustness property ensures reliable causal effect estimation even in the presence of potential misspecification of one of the models.

More specifically, a one-step estimator $\hat{\tau }_{j}$ of the estimand $\tau _{j}$ admits a linear expansion:


\begin{align*} & \hat{\tau}_j - \tau_j = \frac{1}{n}\sum_{i=1}^n \eta_{j}(O_i;\pi,\mu_j) + o_{\mathbb{P}}(n^{-1/2}), \end{align*}

where $\eta _{j}(O_{i};\pi ,\mu _{j})$ is the influence function of $\tau _{j}$, which quantifies how individual observations contribute to the overall estimate. Here, $\pi (\boldsymbol{W}) = \mathbb{P}(A=a \mid \boldsymbol{W})$ is the propensity score model, and $\mu _{j}(\boldsymbol{W},a) = \mathbb{E}[Y_{j} \mid W,A=a]$ is the outcome model for gene $j$.

To estimate the nuisance functions $\mu _{j}$’s (outcome models) and $\pi $ (propensity score model), we use flexible statistical machine learning methods. Specifically, for outcome models $\mu _{j}$, we employ GLMs with a negative binomial likelihood and log link function. This choice accounts for over-dispersion in count data while ensuring computational efficiency given the high dimensionality of genomic data. For the propensity score model $\pi $, we provide two built-in options: (i) logistic regression and (ii) random forests. In our experiments with AD analysis with mixed-type covariates, the random forest model is configured for robustness and performance. Default parameters for each tree include a minimum of 10 samples per leaf, a minimum of 20 samples required to split a node, and the number of features considered for each split set to the square root of the total number of features (max_features=“sqrt”). To account for imbalanced treatment groups, class weights are automatically balanced. Other hyperparameters are then tuned using Extrapolated cross-validation [28] to improve the Gini impurity. Specifically, the number of trees from 200 to 1000, the maximum tree depth is selected from $\{3, 5, 7\}$ and the proportion of samples used to train each tree (max_samples) is selected from $\{0.4, 0.6, 0.8, 1.0\}$. Minimal cost-complexity pruning is also applied with a “ccp_alpha” of $0.02$. Users can also supply alternative estimates for these nuisance functions if desired.

To perform inference, we first compute the estimated influence function values $\hat{\eta }_{j}(O_{i};\hat{\pi },\hat{\mu }_{j})$ and use them to estimate the variance for gene $j$:


\begin{align*} & \hat{\sigma}_j^2 = \frac{\sqrt{n}}{n-1}\sum_{i=1}^n \hat{\eta}_{j}(O_i;\hat{\pi},\hat{\mu}_j)^2. \end{align*}

Using these quantities, a two-sided $t$-statistic for gene $j$ can be computed as: $T_{j} = \frac{\hat{\tau }_{j} - \tau _{j}}{\hat{\sigma }_{j}}.$ This statistic enables hypothesis testing and confidence interval construction for causal effects on gene expression. For further details on the method, see Supplementary Informations S1 and S2.

### Computational considerations

The computational cost of causarray is driven by two components: (i) fitting the generalized factor model to estimate $\widehat U$, and (ii) semiparametric effect estimation and inference across genes. Both components are highly parallelizable across genes, and the memory footprint is dominated by storing the count matrix and low-rank factors.

## Results

### Simulation study demonstrates the advantages of causarray

We evaluate the performance of causarray in two simulated settings (Supplementary Information S3). In the first setting, we generate simulated pseudo-bulk data, while in the second, we generate simulated single-cell data using the Splatter simulator [29], which explicitly models the hierarchical Gamma-Poisson processes underlying scRNA-seq data and captures multi-faceted variability. Each dataset consists of 100–5000 pseudo-bulk or single-cell observations, $\sim $2000 genes, 1 and 2 covariates, and 4 unmeasured confounders.

**Figure 2:**
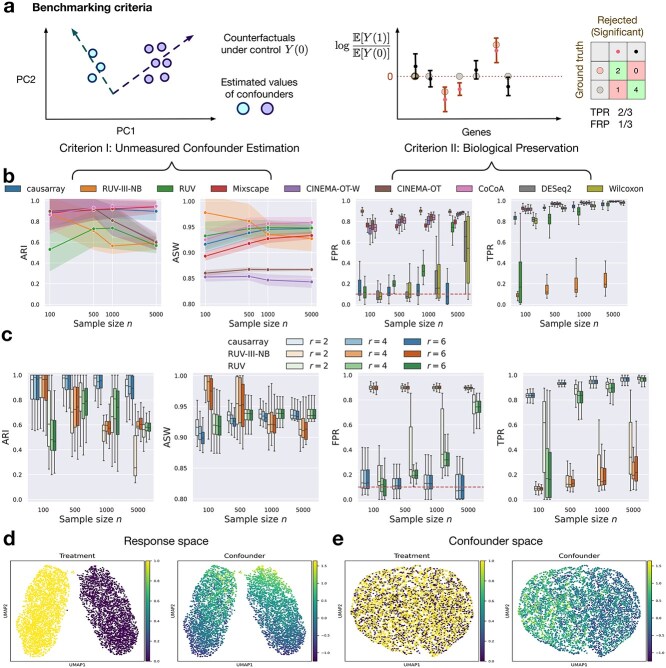
**Benchmarking of causarray against other methods for single-cell DE testing on synthetic expression data with unmeasured confounders.** (a) The analysis pipeline produces a confounder adjustment and a statistic for DE testing. We illustrate two types of criteria used for benchmarking confounder adjustment and DE methods in simulations for pseudo-bulk expressions (b–e) and single-cell (Supplementary Fig. S2) expressions. The metrics for each experimental setting are calculated using 50 simulated datasets with varying random seeds. (b) Performance comparison of causarray and other methods with a well-specified number of latent factors ($r=4$). Line plots show mean ARI and ASW scores for confounder estimation (shaded region represents values within one standard deviation), while box plots display FPR and TPR for biological signal preservation. For the box plots, the center indicates the median, the top and bottom hinges represent the top and bottom quartiles, and the whiskers extend from the hinge to the largest or smallest value no further than 1.5 times the interquartile range from the hinge. (c) Robustness analysis of causarray, RUV-III-NB, and RUV under varying numbers of latent factors ($r=2, 4, 6$). Line plots show mean ARI and ASW scores and box plots show FPR and TPR. (d and e) Causarray disentangles the treatment effects and unmeasured confounding effects in the response and confounder spaces (with $n=5000$ samples). UMAP projection of (d) expression data $Y$ colored by the values of treatment $A$ (purple for control $A=0$ and yellow for treated $A=1$) and unmeasured continuous confounder $U$; and (e) estimated potential outcome under control $Y(0)$ colored by the values of treatment $A$ and continuous confounder $U$.

To benchmark causarray, we compare it with several existing methods designed for DE testing, both with and without confounder adjustment (Fig. 2a). For methods that do not account for unmeasured confounders, we include the Wilcoxon rank-sum test and DESeq2 [30]. In the presence of measured covariates, both regress the gene expression counts with respect to the covariates using the Poisson or negative binomial GLM, respectively. The input to the Wilcoxon rank sum test is the deviance residuals. For confounder-adjusted methods, we consider CoCoA-diff [7], CINEMA-OT [20], CINEMA-OT-W [20], Mixscape [31], RUV [22], and RUV-III-NB [32], where recommended DE test methods are subsequently applied with estimated confounders. RUV estimates latent confounders using RUVSeq [22], followed by DESeq2 [30] for differential testing. RUV-III-NB extends RUVSeq by modeling count data directly using a negative binomial distribution, which improves robustness in overdispersed single-cell RNA-seq data. Mixscape reduces confounding from treatment heterogeneity by using matching to identify and isolate truly responding cells before comparing them to controls for DE analysis. CINEMA-OT employs a heuristic independent space analysis procedure plus optimal transport to separate confounding variables from treatment-associated variables. Compared to CINEMA-OT, CINEMA-OT-W further adjusts for differences in propensity scores between treated and control cells by matching before independent component analysis. A brief summary of each benchmarking comparison method is provided in Supplementary Information S3.2.

To assess the performance of unmeasured confounder adjustment procedures, we use two metrics: adjusted Rand index (ARI) and average silhouette width (ASW). More specifically, we use ARI to quantify the alignment between estimated and true unmeasured confounders and ASW to evaluate cell type separation in the control response space. A higher ARI value indicates better coherence, and a higher ASW value reflects better preservation of biological signals after removing confounding effects. Additionally, to assess the performance of DE testing, we use two metrics: false positive rate (FPR) and true positive rate (TPR) (Supplementary Information S3.3).

We first evaluate how sample size and confounding levels influence the performance of DE testing across methods. Among all tested approaches, only causarray, RUV, Wilcoxon, and DESeq2 effectively control FPR with moderate sample sizes ($n\le 500$) (Fig. 2b and Supplementary Fig. S2a and b). Once the sample size grows larger ($n\!>\!500$), only causarray continues to maintain FPR close to the nominal level of 0.1 across all confounding levels, whereas the FPR of RUV, Wilcoxon, and DESeq2 drifts upward to $\sim 0.15$–$0.22$. On the other hand, Mixscape, CINEMA-OT, CINEMA-OT-W, RUV-III-NB, and CoCoA-diff exhibit inflated FPRs exceeding 0.5 in most cases. Within the subset of methods that control FPR reasonably well (causarray, RUV, Wilcoxon, DESeq2), causarray achieves the highest TPRs, consistently reaching $0.80$–$0.90$ across all scenarios (Fig. 2b). This is significantly higher than DESeq2 and Wilcoxon, particularly for smaller sample sizes in the single-cell experiments (Supplementary Fig. S2a and b). These results highlight causarray’s ability to balance sensitivity and specificity effectively.

In terms of unmeasured confounder adjustment, causarray, RUV-III-NB, and CoCoA-diff achieve both ARI and ASW scores consistently above 0.7 across all sample sizes in both pseudo-bulk and single-cell data (Fig. 2b, Supplementary Fig. S2a and b), outperforming RUV in ARI and CINEMA-OT-W/CINEMA-OT in ASW. Furthermore, causarray effectively disentangles treatment effects from unmeasured confounding effects. In the response space (Fig. 2d), treatment groups are distinctly separated with minimal overlap, while variations within groups reflect unmeasured confounders. In the confounder space (Fig. 2e and Supplementary Fig. S1), causarray produces a uniform mixing of treatment groups while accurately reconstructing continuous confounder values.

Finally, we assess the robustness of causarray, RUV-III-NB, and RUV under varying numbers of latent factors (Fig. 2c and Supplementary Fig. S2c). Among these methods, only causarray consistently controls FPR at nominal levels of 0.1, regardless of the number of factors or sample size. In contrast, RUV-III-NB exhibits inflated median FPRs exceeding 0.2 when more factors are included (e.g. $r = 6$). While RUV-III-NB performs well in terms of ARI (above 0.8) and ASW (above 0.7), its DE testing performance is inferior to RUV due to poor FPR control under certain conditions. Based on these findings, we proceed with causarray and RUV for real data analysis.

### An *in vivo* Perturb-seq study

#### An integrative analysis of multiple single perturbations

ASDs and neurodevelopmental delay (ND) represent a complex group of conditions that have been extensively studied using genetic approaches. To investigate the underlying mechanisms of these disorders, researchers have employed scalable genetic screening with CRISPR-Cas9 technology [26]. Frameshift mutations were introduced in the developing mouse neocortex in utero, followed by single-cell transcriptomic analysis of perturbed cells from the early postnatal brain [26]. These *in vivo* single-cell Perturb-seq data allow for the investigation of causal effects of a panel of ASD/ND risk genes. We analyze the transcriptome of cortical projection neurons (excitatory neurons) perturbed by one risk gene or a non-targeting control perturbation, which serves as a negative control (see Supplementary Information S3.4 for details).

Unmeasured confounders, such as batch effects and unwanted variation, are likely present in this dataset due to the batch design being highly correlated with perturbation conditions (Supplementary Fig. S3a and b). Additionally, the heterogeneity of single cells assessed *in vivo* introduces further complexity. These confounding factors may reduce statistical power for gene-level DE tests, as noted in the original study [26], which instead focused on gene module-level effects. To address this limitation, we apply causarray to incorporate unmeasured confounder adjustment and conduct a more granular analysis at the single-gene level. This approach enables us to uncover nuanced genetic interactions and causal effects that may provide deeper insights into the etiology of ASD/ND.

#### Functional analysis

Gene module-level analyses have been shown to provide greater statistical power for detecting biologically meaningful perturbation effects when fewer cells are available [26]. The original study adopted this approach but relied on a linear model rather than a negative binomial model, potentially limiting its ability to detect broader signals at the individual gene level. Here, we compare causarray with RUV and DESeq2 (without confounder adjustment) to identify significant genes and enriched gene ontology (GO) terms associated with various perturbations. The number of latent factors is set as 10, according to the joint-likelihood-based information criterion (Supplementary Information S1.3, Supplementary Fig. S4a).

**Figure 3:**
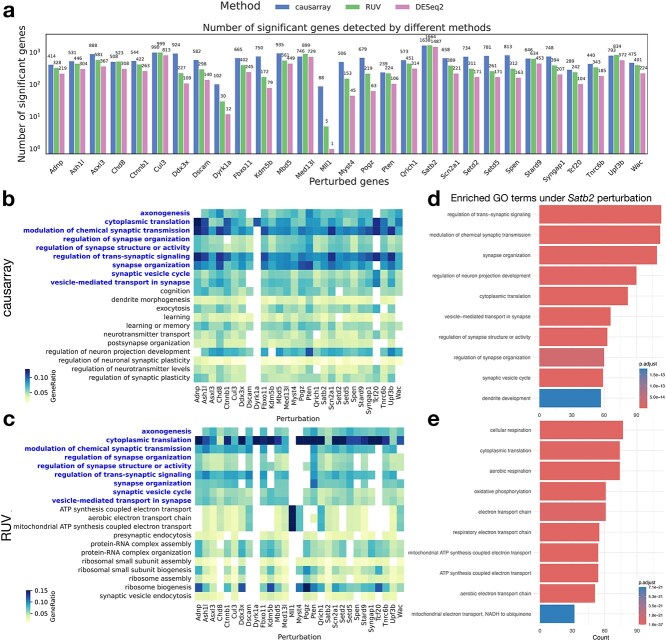
**Comparison of DE genes discovered by causarray and RUV on excitatory neurons for AD.** (a) The ratio of false discoveries to all 15586 genes of DE test results with permuted disease labels on the ROSMAP-AD dataset. Three methods, causarray with FDX control, causarray with FDR control, and RUV with FDR control, are compared. Data are presented as mean values $\pm $ s.d. (b) The similarity of estimated effect sizes on SEA-AD MTG and PFC datasets. The slope is estimated from linear regression of effect sizes on the PFC dataset against those on the MTG dataset. (d) Considering only the top 50 positively regulated and the top 50 negatively regulated DE genes from causarray and RUV, we map them to the top 5 biological processes (the green nodes). (e) Estimated counterfactual distributions of 10 selected genes by causarray. The top 5 up-regulated and top 5 down-regulated genes in estimated LFCs (adjusted $P$ value $<0.05$) are visualized. The values are displayed on a log scale after adding a pseudo-count of one. (f) Estimated log-fold change of treatment effects, conditional on age for selected genes. The center lines represent the mean of the locally estimated scatter plot smoothing (LOESS) regression, and the shaded area represents a 95% confidence interval at each value of age.

In terms of significant gene detection, causarray identifies a comparable number of significant genes to RUV across most perturbations, while DESeq2 consistently detects fewer significant genes (Fig. 3a). The variation in significant detections across different perturbed genes suggests distinct biological impacts of each knockout. Functional analysis focuses on enriched GO terms on the DE genes under each perturbation condition where discrepancies arise between causarray and other methods. Genes identified by causarray are enriched for biologically relevant GO terms with clear clustering patterns (Fig. 3b and c, Supplementary Fig. S3c). In contrast, RUV shows less distinct clustering and enrichment patterns.

**Figure 4:**
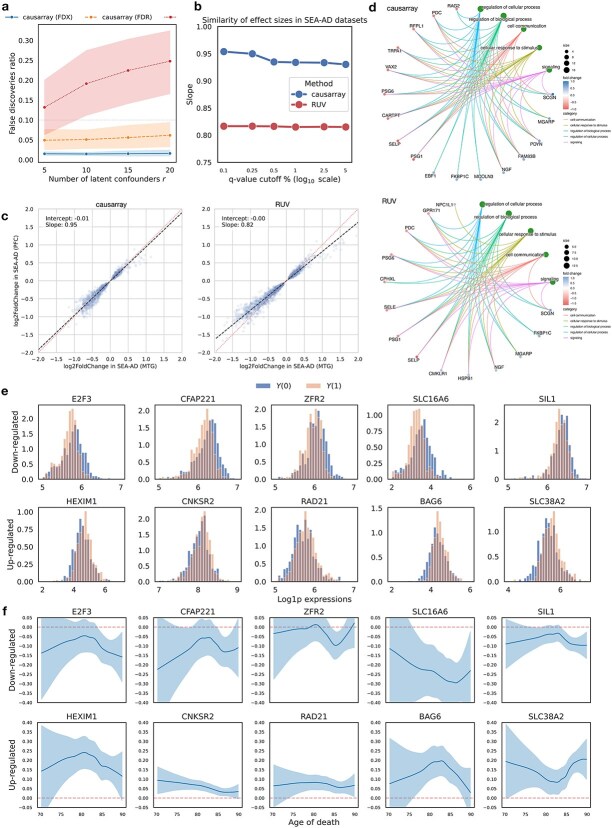
**Statistical test results of the effects of CRISPR perturbation on gene expression in excitatory neuron data.** (a) Number of significant genes detected under all perturbations using three different methods. The detection threshold for significant genes is FDR$ < 0.1$ for all methods. (b and c) Heatmaps of GO terms enriched (adjusted $P$ value $<0.05$, $q<0.2$) in discoveries from causarray and RUV, respectively, where the common GO terms are highlighted in blue. Only the top 20 GO terms that have the most occurrences in all perturbations are displayed. (d and e) Barplots of GO terms enriched in discoveries under *Satb2* perturbation from causarray and RUV, respectively.

Notably, while RUV identifies GO terms related to ribosome processes previously implicated in ASD studies [33], these findings remain controversial. Some argue that dysregulation in translation processes and ribosomal proteins may reflect secondary changes triggered by expression alterations in synaptic genes rather than direct causal effects [34]. In contrast, GO terms identified by causarray align more closely with the expected causal effects of ASD/ND gene perturbations [35, 36].

To further validate these findings, we examine the perturbation condition for *Satb2*, which yields the largest number of significant genes identified by both methods (adjusted $P$ value $<.1$) and exhibits significantly different estimated propensity scores (Supplementary Fig. S4b). *Satb2* is known to play critical roles in neuronal development, synaptic function, and cognitive processes [37, 38]. Using causarray, we detect enrichment for GO terms directly related to neuronal function and development, such as “regulation of neuron projection development,” “regulation of synapse structure or activity,” and “synapse organization” (Fig. 3d). These findings are consistent with *Satb2*’s established roles in neuronal development and synaptic plasticity [39, 40]. On the other hand, RUV identifies enrichment for terms related to mitochondrial function and energy metabolism, such as “mitochondrial electron transport,” “cellular respiration,” and “ATP synthesis” (Fig. 3e). While these processes are important for general cellular function, they are less directly relevant to *Satb2*’s primary biological roles. For an extended list of GO terms, see Supplementary Fig. S3c.

A gene-level breakdown under *Satb2* (including causarray-only genes in GO:0021953 and their association with the estimated latent factors $\hat U$) is provided in Supplementary Information S3.4 (Supplementary Fig. S5a and b). We additionally applied ComBat [41] as a representative design-based correction method that requires known batch labels. In the *Satb2* perturbation, ComBat identifies 1710 DE genes, but yields no GO biological process terms passing multiple-testing correction (adjusted $P<.05$), whereas causarray identifies 1638 DE genes and RUV identifies 1705 (FDR $<0.1$) (Supplementary Fig. S5c and d). Moreover, ComBat’s top GO terms are broad, high-level categories (e.g. signaling, phosphorylation, cell adhesion), while *Satb2* knockdown is expected to enrich neuronal development and synaptic programs. This illustrates a practical failure mode when batch design is strongly aligned with perturbation assignment: design-based correction can reduce biological specificity, motivating causal estimation procedures that explicitly model treatment assignment and latent confounding.

Overall, this analysis demonstrates that causarray provides greater specificity in detecting biologically meaningful causal effects of gene perturbations. Its ability to disentangle confounding influences while preserving relevant biological signals highlights its effectiveness in analyzing complex genomic datasets.

### Alzheimer’s disease case-control study

#### An integrative analysis of excitatory neurons

We analyze three AD single-nucleus RNA sequencing datasets: a transcriptomic atlas from the Religious Orders Study and Memory and Aging Project (ROSMAP) [42] and two datasets from the Seattle Alzheimer’s Disease Brain Cell Atlas (SEA-AD) consortium [43], which include samples from the middle temporal gyrus (MTG) and prefrontal cortex (PFC). Our objective is to compare the performance of causarray and RUV in pseudo-bulk DE tests of AD in excitatory neurons (see Supplementary Information S3.5 for details).

To evaluate the validity, we perform a permutation experiment on the ROSMAP-AD dataset by permuting phenotypic labels. Ideally, no significant discoveries should be made under this null scenario. However, RUV produces a large number of false discoveries, with its performance deteriorating as the number of latent factors increases. In contrast, causarray effectively controls the false discovery rate (FDR), producing minimal false positives (Supplementary Fig. S6a), and the results using FDX are conservative. Additionally, we assess coherence across datasets by examining effect sizes in SEA-AD (MTG) and SEA-AD (PFC). Because we cannot know the ground truth, our objective is to assess the stability of results across the three related studies. Effect sizes estimated by causarray exhibit higher consistency across varying q-value cutoffs compared to RUV (Supplementary Fig. S6b and c). We further compare functional enrichment results between causarray and RUV using GO. When inspecting DE genes across all three AD datasets, causarray identifies similar numbers of discoveries as RUV, and 1186 terms were associated with them (Supplementary Information S3.5, Supplementary Fig. S6b). The discovered networks, as defined as the top 5 GO terms and associated genes included in the top 100 DE gene discoveries, show the enhanced sensitivity and comprehensiveness of causarray (Supplementary Fig. S6d).

#### Counterfactual analysis

The counterfactual framework employed by causarray enables downstream analyses that directly utilize estimated potential outcomes. By examining counterfactual distributions for significant genes (Supplementary Fig. S6a), we observe distinct shifts in expression levels between treatment ($Y(1)$) and control ($Y(0)$) groups. Downregulated genes show a shift toward lower expression levels under disease conditions, while upregulated genes exhibit increased expression. Conditional average treatment effects (CATEs) reveal age-dependent trends for these genes (Supplementary Fig. S6b). For example, upregulated genes such as *SLC16A6* and *HEXIM1* show stronger effects at extreme ends of the age distribution, while others like *SLC38A2* and *BAG6* display nuanced changes across the aging spectrum.

These findings align with prior studies highlighting the roles of specific genes in aging-related processes. For instance, *ZFR2*, *SIL1*, *BAG6*, and *RAD21* have been implicated in chromatin remodeling, synaptic plasticity, and cellular stress responses critical for aging and neurodegeneration [44–47]. While nonparametric fitted curves exhibit wider uncertainty bands, particularly at the boundaries, which can be observed here, the significant trends observed for key genes highlight their potential relevance in AD pathology. Overall, these results demonstrate that causarray provides nuanced insights into age-dependent gene regulation mechanisms while maintaining robust control over confounding influences.

## Conclusion

The rapid growth of high-throughput single-cell technologies has created an urgent need for robust causal inference frameworks capable of disentangling treatment effects from confounding influences. Existing methods, such as CINEMA-OT [20], have advanced the field by separating confounder and treatment signals and providing per-cell treatment-effect estimates. However, these methods rely on the assumption of no unmeasured confounders, which is often violated in observational studies and *in vivo* experiments. Additionally, many confounder adjustment methods, such as RUV [22], depend on linear model assumptions that do not directly model count data or provide robust DE testing at the gene level. Similarly, popular single-cell integration tools primarily target cross-batch alignment in a transformed or embedded space rather than producing corrected count-scale expression for DE testing (Supplementary Information S1.6). Likewise, while DESeq2 [30] is a widely used tool for DE analysis of count data, it assumes that treatment assignment is unconfounded after accounting for observed covariates and does not explicitly model latent confounding. In contrast, causarray builds on the generalized linear modeling framework to estimate unmeasured confounders and extend the standard parametric models to a semiparametric regime with flexible nuisance function estimation. This distinction is critical in single-cell and case–control studies where hidden batch or biological effects can otherwise bias DESeq2’s inference.

Causarray directly models count data using GLMs for unmeasured confounder estimation, overcoming a key limitation of RUV in DE analysis. Unlike CINEMA-OT [20] and CoCoA-diff [7], which rely on optimal transport or matching techniques, causarray employs a semiparametric framework that combines flexible machine learning models with semiparametric inference. This approach enhances stability and interpretability while enabling valid statistical inference of treatment effects. Benchmarking results demonstrate that causarray outperforms existing methods in disentangling treatment effects from confounding influences across diverse experimental settings, maintaining superior control over false positive rates while achieving higher true positive rates. The use of GLMs allows more accurate estimation of latent confounders in sparse, overdispersed count data, improving the robustness of confounder adjustment relative to linear or heuristic matching approaches. By explicitly modeling the mean–variance relationship of single-cell data, the GLM formulation helps extract biologically relevant latent structure that would otherwise be absorbed as noise. Nevertheless, errors in the estimated confounders $\hat{U}$ can propagate into downstream causal estimates, leading to bias or inflated variance when the latent structure is weakly identifiable. Accordingly, causal interpretation in causarray is conditional on standard identification assumptions (consistency, conditional exchangeability given $(X,\widehat U)$, and positivity) and on the adequacy of the estimated latent structure for capturing residual shared variation relevant to treatment assignment and expression. When overlap is limited, or the latent structure is weakly identifiable, estimates can be sensitive to modeling choices (including $r$) and should be interpreted as robustness-focused causal evidence under assumptions rather than as unconditional causal ground truth. These effects are inherent to all confounder-adjustment approaches and underscore the importance of diagnostic checks, such as overlap assessment and sensitivity analyses, to evaluate the robustness of causal conclusions.

In an *in vivo* Perturb-seq study of ASD/ND genes, causarray uncovered gene-level perturbation effects that were missed by prior module-based analyses. The Perturb-seq experiment involved 30 distinct perturbation conditions, all analyzed jointly by causarray within a unified framework that adjusts for shared confounding across groups. It identified biologically relevant pathways linked to neuronal development and synaptic functions for multiple autism risk genes. Similarly, in a case-control study of AD using three human brain transcriptomic datasets, causarray revealed consistent causal gene expression changes across datasets and highlighted key biological processes such as synaptic signaling and cell development. The same framework is readily extensible to multi-group comparisons in disease studies-e.g. different stages of Alzheimer’s progression—allowing stage-specific effect estimation while accounting for shared sources of variation. These findings underscore the ability of causarray to provide biologically meaningful insights across diverse contexts.

Despite its strengths, causarray has certain limitations. Its performance depends on the accurate estimation of unmeasured confounders, which may vary with dataset complexity and experimental design. Furthermore, while causarray provides robust DE testing, its integration with advanced spatial or trajectory analysis frameworks remains unexplored [48, 49]. Future research could focus on extending causarray to incorporate prior biological knowledge or extrapolate to unseen perturbation-cell pairs, similar to emerging methods like CPA [50]. Such advancements would further enhance its applicability in single-cell causal inference on general omics.

Finally, in this study, we focus our analysis on scRNA-seq outcomes, but emerging technologies now enable a wide range of omic readouts. In the perturbation framework, these include chromatin-based readouts with Perturb-ATAC [51], protein-level profiling with ProCODE [52], and joint RNA–protein measurements with ECCITE-seq [53]. causarray can easily be extended to any of these readouts.

**Key Points**

- We introduce causarray, a causal inference framework for single-cell and pseudo-bulk omics that couples generalized confounder adjustment for count data with semiparametric inference.
- In simulations, causarray disentangles treatment effects from unmeasured confounding, maintains nominal false-positive control, and achieves higher power while preserving biological structure across sample sizes and latent-factor choices.
- In a benchmarking study, causarray performs better than other methods, both in terms of confounder estimation and testing performance (true positives and true negatives).
- Applied to *in vivo* Perturb-seq of autism spectrum disorder/neurodevelopmental disorder genes, causarray detects gene-level causal effects enriched for neuronal development and synaptic pathways, offering greater specificity than alternative methods.
- In Alzheimer’s case-control analyses spanning Religious Orders Study and Memory and Aging Project and Seattle Alzheimer’s Disease Brain Cell Atlas (SEA-AD) cohorts, causarray yields reproducible effect sizes, rigorous error-rate control, and clear counterfactual shifts in expression that highlight synaptic and cell-development processes.
- By estimating per-gene potential outcomes, causarray enables downstream counterfactual and conditional treatment-effect analyses (for example, age-dependent trends), providing a practical route from association toward causal effect estimation under explicit identification assumptions.

## Supplementary Material

supp_bbag175

## Data Availability

All datasets used in this paper are previously published and freely available, except the metadata for donors from the ROSMAP cohort. The Perturb-seq dataset is available through the Broad single cell portal as txt files. The gene expression count matrices of ROSMAP-AD datasets [42] can be obtained from supplementary website, which have been deidentified to protect confidentiality—the mapping to ROSMAP IDs and complete metadata can be found on Synapse as Seurat objects (rds files). The SEA-AD datasets of nuclei-by-gene matrices with counts and normalized expression values from the snRNA-seq assay [43] are available through the Open Data Registry in an AWS bucket (sea-ad-single-cell-profiling) as AnnData objects (h5ad files). The code for reproducing the results in the paper and the causarray package can be accessed at https://github.com/jaydu1/causarray.
