# Supplementary material for: Uncovering causal relationships in single-cell omic studies with causarray
Source: Brief Bioinform. 2026 Apr 15;27(2):bbag175. doi: 10.1093/bib/bbag175 (PMC13082396; doi:10.1093/bib/bbag175)
Supplement: supp_bbag175 [file supp_bbag175.pdf]

Supplementary Information S1: Confounder estimation

S1.1 Unit of analysis and dependence

When many cells are sampled from the same donor/subject, within-donor correlation violates an i.i.d. cell-level assumption; in such settings, we recommend donor-level pseudo-bulk aggregation and treat donors as the experimental units (as in our Alzheimer's disease analyses). Cell-level analyses are most appropriate when cells are approximately independent experimental units (e.g., cell lines or weakly clustered designs).

S1.2 Algorithm

To estimate the unmeasured confounders, we employ an improved version of GCATE (6). Suppose (X_i, A_i, Y_i) for $i = 1, \dots, n$ are n independently and identically distributed samples coming from the same distribution as $(X, A, Y) \in \mathbb{R}^d \times \mathbb{R}^a \times \mathbb{R}^p$. Here, A consists of a treatments and can be both continuous and discrete for the purpose of confounder estimation. Let $\boldsymbol{X} \in \mathbb{R}^{n\times d}, \boldsymbol{A} \in \mathbb{R}^{n\times a}, \boldsymbol{Y} \in \mathbb{R}^{n\times p}$ denote the design matrix, treatment matrix, and gene expression matrix, respectively. To account for different library sizes, we model the mean of the size-normalized counts

$$\mu_{ij} = \frac{Y_{ij}}{s_i},$$

which is assumed to follow a negative binomial distribution. Technically, μ_{ij}'s should be non-negative integers; however, the likelihood-based approaches work seamlessly even when they are non-negative real numbers. Here s_i is the size factor of cell i, which will be specified later. We assume the conditional mean is characterized by a generalized linear model

$$\log \mu_{ij} \sim A_i + X_i + U_i,$$

and its dispersion parameter ϕ is predetermined.

The adjusted expression μ_{ij} of the ith observation and the jth gene has the density:

$$p(\mu_{ij} \mid \theta_{ij}) = h(\mu_{ij}) \exp\left(\mu_{ij}\theta_{ij} - A(\theta_{ij})\right),$$

where θ_{ij} is the natural parameter. In matrix form, the natural parameters decompose as

$$\boldsymbol{\Theta} = \widetilde{\boldsymbol{X}}\boldsymbol{B}^\top + \boldsymbol{U}\boldsymbol{\Gamma}^\top,$$

where $\widetilde{\boldsymbol{X}} = [\boldsymbol{X}, \boldsymbol{A}] \in \mathbb{R}^{n\times(d+a)}$, $\boldsymbol{B} \in \mathbb{R}^{p\times(d+a)}$, $\boldsymbol{U} \in \mathbb{R}^{n\times r}$, and $\boldsymbol{\Gamma} \in \mathbb{R}^{p\times r}$ are unknown. Note that μ_{ij}'s are conditionally independent given the natural parameter $\boldsymbol{\Theta}$. Under such a model, the theoretical foundations for identifying U are provided in prior work (6), which establishes conditions under which $\boldsymbol{U}$ can be consistently estimated from observed data using matrix factorization techniques, even in the presence of latent confounding. With this notation, the procedure of unmeasured confounder estimation is summarized in Algorithm S1, and the details of the method are described below.

Estimation of size factors.. We follow the procedure in (15) to compute the size factors s_i for $i = 1, \dots, n$. We start by calculating the geometric mean for each gene j:

$$g_j = \exp\left(\frac{\sum_i \log(Y_{ij})\mathbb{1}\{Y_{ij} > 0\}}{\sum_i \mathbb{1}\{Y_{ij} > 0\}}\right).$$

Next, for each sample i, compute the initial size factors:

$$d_i = \exp\left(\underset{j:Y_{ij}>0}{\text{median}}\{\log(Y_{ij}) - \log(g_j)\}\right).$$

Finally, we normalize these size factors to have a geometric mean of 1 across all samples:

$$s_i = \frac{d_i}{(\prod_i d_i)^{1/n}}. \tag{S1}$$

The size factors can then be used to normalize gene expression data, adjusting for differences in sequencing depth and other systematic biases across samples. The normalization ensures that observed differences in expression levels reflect true biological variation rather than technical artifacts.

Algorithm S1 Unmeasured confounder estimation

Input: A data matrix $\boldsymbol{Y} \in \mathbb{R}^{n\times p}$, a design matrix $\widetilde{\boldsymbol{X}} = [\boldsymbol{X}, \boldsymbol{A}] \in \mathbb{R}^{n\times(d+a)}$, a natural number $r \geq 1$ (the number of latent factors), a constant $C = 2\times 10^3$ for the norm constraint

1: (Estimation of size factors) Compute $\boldsymbol{s} \in \mathbb{R}^n$ according to Eq. (S1).
2: (Estimation of dispersion parameters) Compute $\boldsymbol{\phi} \in \mathbb{R}^p$ according to Eq. (S2).
3: (Estimation of marginal effects $\boldsymbol{F}$ and uncorrelated latent components $\boldsymbol{W}\boldsymbol{\Gamma}^\top$) Solve optimization problem Eq. (S3) to obtain $\widehat{\boldsymbol{W}}_0\widehat{\boldsymbol{\Gamma}}_0^\top$ and the initial estimate of the natural parameter matrix $\widehat{\boldsymbol{\Theta}}_0 = \widetilde{\boldsymbol{X}}\widehat{\boldsymbol{F}}^\top + \widehat{\boldsymbol{W}}_0\widehat{\boldsymbol{\Gamma}}_0^\top$ by alternative maximization:

$$\begin{aligned} \widehat{\boldsymbol{F}}, \widehat{\boldsymbol{W}}_0, \widehat{\boldsymbol{\Gamma}}_0 \in & \operatorname*{argmin}_{\boldsymbol{F}\in\mathbb{R}^{p\times(d+a)}, \boldsymbol{W}\in\mathbb{R}^{n\times r}, \boldsymbol{\Gamma}\in\mathbb{R}^{p\times r}} \mathcal{L}(\widetilde{\boldsymbol{X}}\boldsymbol{F}^\top + \boldsymbol{W}\boldsymbol{\Gamma}^\top) \\ \text{subject to} \quad & \widetilde{\boldsymbol{X}}\boldsymbol{F}^\top + \boldsymbol{W}\boldsymbol{\Gamma}^\top \in \mathcal{B}_C^{n\times p}, \qquad \mathcal{P}_{\widetilde{\boldsymbol{X}}}\boldsymbol{W} = \boldsymbol{0}. \end{aligned} \tag{S3}$$

4: (Estimation of latent coefficients $\boldsymbol{\Gamma}$) Set $\widehat{\boldsymbol{W}} := \sqrt{n}\boldsymbol{Q}\boldsymbol{\Sigma}^{1/2}$ and $\widehat{\boldsymbol{\Gamma}} := \sqrt{p}\boldsymbol{V}\boldsymbol{\Sigma}^{1/2}$, where $\widehat{\boldsymbol{W}}_0\widehat{\boldsymbol{\Gamma}}_0^\top = \sqrt{np}\boldsymbol{Q}\boldsymbol{\Sigma}\boldsymbol{V}^\top$ is the condensed SVD with $\boldsymbol{Q} \in \mathbb{R}^{n\times r}$, $\boldsymbol{\Sigma} \in \mathbb{R}^{r\times r}$, $\boldsymbol{V} \in \mathbb{R}^{p\times r}$.
5: (Estimation of direct effects $\boldsymbol{B}$ and latent factors $\boldsymbol{U}$) Solve optimization problem Eq. (S4) to obtain $(\widehat{\boldsymbol{B}}, \widehat{\boldsymbol{U}})$:

$$\begin{aligned} \widehat{\boldsymbol{B}}, \widehat{\boldsymbol{U}} = & \operatorname*{argmin}_{\boldsymbol{B}\in\mathbb{R}^{p\times(d+a)}, \boldsymbol{U}\in\mathbb{R}^{p\times r}} \mathcal{L}(\widetilde{\boldsymbol{X}}\boldsymbol{B}^\top + \boldsymbol{U}\widehat{\boldsymbol{\Gamma}}^\top) + \sum_{j=1}^p \lambda_j \|\boldsymbol{B}_{(d+1):(d+a),j}\|_1 \\ \text{subject to} \quad & \widetilde{\boldsymbol{X}}\boldsymbol{B}^\top + \boldsymbol{U}\widehat{\boldsymbol{\Gamma}}^\top \in \mathcal{B}_C^{n\times p}, \qquad \mathcal{P}_{\widehat{\boldsymbol{\Gamma}}}\boldsymbol{B} = \boldsymbol{0}. \end{aligned} \tag{S4}$$

Output: Return the estimated confounders $\widehat{\boldsymbol{U}}$.

Estimation of dispersion parameters.. To estimate the dispersion parameter, we first fit generalized linear models (GLMs) on the data and obtain the estimated mean expression of gene j, denoted as $\widehat{\nu}_j$ for $j = 1,\ldots,p$. Note that when μ_{ij} comes from a Negative Binomial distribution, its variance is given by

$$\mathrm{Var}(\mu_{ij} \mid \theta_{ij}) = \nu(1+\alpha_j\nu),$$

where $\nu = \mathbb{E}[\mu_{ij} \mid \theta_{ij}]$ is the conditional mean while α_j is the dispersion parameter of the NB1 form. In the form of an exponential family parameterized by the parameter ϕ_j, α_j is the reciprocal of ϕ_j, namely, $\alpha_j = 1/\phi_j$. By methods of moments, we can solve the following equation to obtain an estimator $\widehat{\phi}_j$ for ϕ_j:

$$\frac{1}{n}\sum_{i=1}^n (y_{ij} - \widehat{\nu}_j)^2 = \widehat{\nu}_j(1+\alpha\widehat{\nu}_j).$$

Finally, we clip $\widehat{\alpha}_j$ to be in $[10^{-2}, 10^2]$ and set $\widehat{\phi}_j = 1/\widehat{\alpha}_j$. The estimated dispersion parameter has a closed-form expression:

$$\phi_j = \min\left\{\max\left\{\frac{\widehat{\nu}_j^2}{\frac{1}{n}\sum_{i=1}^n (y_{ij}-\widehat{\nu}_j)^2 - \widehat{\nu}_j}, 0.01\right\}, 100\right\}. \tag{S2}$$

Estimation of marginal effects by joint likelihood estimation.. The negative log-likelihood function of the data is given by

$$\mathcal{L}(\boldsymbol{\Theta}) = \mathcal{L}(\boldsymbol{B}, \boldsymbol{U}, \boldsymbol{\Gamma}) = -\frac{1}{n}\sum_{i=1}^n\sum_{j=1}^p \left(\mu_{ij}\theta_{ij} - A(\theta_{ij}) + \log\binom{\mu_{ij}+\phi_j-1}{\mu_{ij}}\right).$$

Although this is a nonconvex optimization problem, an alternative descent algorithm as in (6) can be employed to solve it efficiently. By rewriting $\boldsymbol{\Theta} = \widetilde{\boldsymbol{X}}\boldsymbol{B}^\top + \boldsymbol{Z}\boldsymbol{\Gamma}^\top$ as $\boldsymbol{\Theta} = \widetilde{\boldsymbol{X}}\boldsymbol{F}^\top + \boldsymbol{W}\boldsymbol{\Gamma}^\top$ with $\mathcal{P}_{\widetilde{\boldsymbol{X}}}\boldsymbol{W} = \boldsymbol{0}$, we can disentangle the marginal effects and the uncorrelated latent components. This corresponds to step 3 of Algorithm S1. Each entry of the estimated natural parameter matrix is constrained within the Euclidean ball $\mathcal{B}_C$ with radius C ($C = 2\times 10^3$ by default).

Before alternative maximization, we compute deviance residuals $\boldsymbol{R}$ from the NB GLM fits with offsets $\log\boldsymbol{s}$ and dispersion parameters ϕ, and initialize the uncorrelated confounders by $\boldsymbol{W} = \mathcal{P}_{\widetilde{\boldsymbol{X}}}^\perp \boldsymbol{U}_{\boldsymbol{R}}$ where $\boldsymbol{U}_{\boldsymbol{R}} \in \mathbb{R}^{n\times r}$ contains the first r left singular vectors of $\boldsymbol{R}$. Here, the projection $\mathcal{P}_{\widetilde{\boldsymbol{X}}}^\perp$ ensures that $\boldsymbol{W}$ is uncorrelated with $\boldsymbol{X}$. Then, we initialize the marginal effects $\boldsymbol{F}$ and latent coefficient $\boldsymbol{\Gamma}$ by solving GLMs with covariates $[\widetilde{\boldsymbol{X}}, \boldsymbol{W}]$. In particular, when the intercept is included in the covariates, the initial value of $\boldsymbol{W}$ also has zero means per column.

Estimation of latent coefficients.. Because the (uncorrelated) latent factors are identifiable only up to scaling and rotations, we rescle the estimate at step 4 of Algorithm S1. This ensures the eigenvalues of $\widehat{\boldsymbol{W}}$ and $\widehat{\boldsymbol{\Gamma}}$ have the same order, making the alternative optimization more stable.

Estimation of confounding effects by adaptive penalization.. The last step is to jointly recover the direct effects and the unmeasured confounders. This is done by imposing orthogonality between $\widehat{\boldsymbol{B}}$ and $\widehat{\boldsymbol{\Gamma}}$, as well as imposing sparsity on $\widehat{\boldsymbol{B}}$. The former ensures the gene-wise effects of the observed covariates and the unmeasured confounders are uncorrelated, while the latter aims to reveal signals from noisy measurements.

The direct effect $\boldsymbol{B}$ is initialized as $\mathcal{P}^{\perp}_{\widehat{\boldsymbol{\Gamma}}}\widehat{\boldsymbol{F}}$. Then, Initialize $\boldsymbol{Z}$ and $\boldsymbol{\Gamma}$ using the SVD of the matrix $\boldsymbol{X}\widehat{\boldsymbol{F}}^{\top}\mathcal{P}_{\widehat{\boldsymbol{\Gamma}}} + \widehat{\boldsymbol{W}}\widehat{\boldsymbol{\Gamma}}^{\top} = \boldsymbol{U}'\boldsymbol{\Sigma}'\boldsymbol{V}'^{\top}$. Let $\boldsymbol{Z} = (\boldsymbol{U}'\boldsymbol{\Sigma}'^{1/2})_{1:r}$ and $\boldsymbol{\Gamma} = (\boldsymbol{V}'\boldsymbol{\Sigma}'^{1/2})_{1:r}$ be the initialized values.

To account for different scales of the effects induced by different treatment conditions, we propose to use the adaptive lasso to induce sparsity of effects from multiple treatments. The coefficients for non-treatment covariates $\boldsymbol{X}$ are not penalized in our implementation. Only the treatment-related coefficients for $\boldsymbol{A}$ are subject to penalization. More specifically, in optimization problem S4, the regularization parameters are set as

$$\lambda_j = \lambda / \|(\mathcal{P}^{\perp}_{\widehat{\boldsymbol{\Gamma}}}\widehat{\boldsymbol{F}})_{\cdot j}\|_1, \qquad j = 1, \ldots, p$$

where $\widehat{\boldsymbol{F}}$ is the design matrix and $\mathcal{P}^{\perp}_{\widehat{\boldsymbol{\Gamma}}}$ projects out estimated confounding effects. This scaling adjusts for the varying magnitudes across genes and ensures that the penalization is adaptive to gene-specific signal strength, in contrast to using a single global λ as in GCATE (6). In our implementation, we use a default value of $\lambda = 0.05$, which was chosen based on empirical performance across several datasets. Results are not overly sensitive to small variations in λ due to the gene-wise normalization. Users can also tune λ via cross-validation if needed.

Because of regularization, the estimate $\widehat{\boldsymbol{B}}$ is typically biased towards zero, so we don't use it for downstream analysis. It is possible to perform inference with an additional debiasing procedure (6). However, we use a more flexible semiparametric inference method, as described below in the next section.

S1.3 Determine the number of latent factors r

To determine the number of unmeasured confounders r, one can use the joint-likelihood-based information criterion (JIC) (6). The JIC value is the sum of deviance and a penalty on model complexity:

$$\mathrm{JIC}(\widehat{\boldsymbol{\Theta}}^{(r)}) = -2\sum_{i=1}^{n}\sum_{j=1}^{p}\log p(\mu_{ij} \mid \widehat{\theta}_{ij}^{(r)}) + c_{\mathrm{JIC}} \cdot \frac{(d+a+r)\log(n \wedge p)}{n \wedge p},$$

where $\widehat{\boldsymbol{\Theta}}^{(r)}$ is the estimated natural parameter matrix with r unmeasured confounders and $d+a$ observed covariates, and $c_{\mathrm{JIC}} > 0$ is a universal constant set to be 1 by default.

Because "no unmeasured confounding" is not testable from observed data alone, we cannot certify that a dataset is unconfounded, but can provide actionable diagnostics that (a) flag when confounding is likely and (b) summarize how much it could matter for inference. This can be achieved by estimating the latent structure to quantify residual shared variation not explained by X, and summarize severity by the magnitude of change in gene-level effects when adjusting for $(X, \widehat{U})$ versus X alone (e.g., correlation or median absolute change of $\widehat{\beta}$ across genes). If the estimated number of latent factors is near zero and effect estimates are essentially unchanged after including $\widehat{U}$, this provides evidence that residual unmeasured confounding beyond X is limited (while not constituting a proof of absence).

S1.4 Comparison with reference-based confounder adjustment methods

Another approach to adjust for the unmeasured confounders is to utilize the information from negative control genes. This includes scMerge (13), RUV-III-NB (23) and RUVSeq (22) etc. These methods require users to specify a set of negative control genes, such as housekeeping genes, which are assumed to be solely due to unwanted variation between the two cells. The approach necessitates strong prior knowledge to accurately identify negative control genes, which may not always be available, especially in less well-characterized biological systems. This reliance on prior knowledge can limit the applicability of the method in novel or poorly understood contexts.

S1.5 Comparison with RUV and RUV-III-NB

The primary reason for RUVr's poor performance in this causal inference setting is its two-stage estimation strategy. RUVr first removes the variation associated with treatment A (and other observed covariates) and then searches for latent factors in the remaining residual variation. By design, this procedure identifies factors of unwanted variation that are orthogonal to treatment A. This is fundamentally at odds with the goal of adjusting for confounding in observational studies. A confounder U is, by

definition, a variable that influences both the treatment A and the outcome Y. RUVr's approach systematically fails to estimate any part of a latent factor that is correlated with the treatment, leading to underestimated variance and inflated Type I error.

This conceptual limitation is compounded by a model mismatch. RUVr assumes a linear model, which is ill-suited for the sparse, non-normal count data characteristic of single-cell sequencing even after log-transformation. In contrast, our method utilizes a more appropriate GLM framework and estimates effects iteratively, allowing it to identify confounders that are correlated with the treatment.

S1.6 Relationship to data integration

Popular data integration methods aim to align datasets in a shared low-dimensional space to improve downstream tasks like clustering, visualization, and label transfer (5). These objectives differ from causal effect estimation: integration optimizes removal of variation that impedes cross-batch mixing, whereas causal DE requires adjusting for confounding that is correlated with treatment while preserving the treatment signal. When batch structure (or other unwanted variation) is aligned with treatment assignment, "removing batch" can also remove part of the treatment effect, so integration success metrics need not correspond to unbiased gene-level effects. In contrast, causarray treats technical and biological unwanted variation as potential confounding and adjusts for it through a generalized factor model coupled with doubly robust estimation, producing gene-wise causal estimands with valid uncertainty quantification under stated identification assumptions.

Supplementary Information S2: Statistical inference

S2.1 Counterfactual

Potential outcomes framework. Let $O = (A, W, Y) \in \{0,1\} \times \mathbb{R}^{d_W} \times \mathbb{R}^p$ be a tuple of random vectors, where A is the binary treatment variable (e.g., presence or absence of a disease or perturbation), W is the vector of covariates (e.g., biological or technical factors influencing both treatment and outcome), and Y is the observed outcomes, defined as $Y = AY(1) + (1-A)Y(0)$, where $Y(1)$ and $Y(0)$ are the potential outcomes under treatment and control, respectively.

The potential outcomes framework assumes that for each individual or observation, there exist two potential outcomes: one if the individual receives the treatment ($Y(1)$) and one if they do not ($Y(0)$). However, only one of these outcomes can be observed for each individual, depending on whether they were treated ($A = 1$) or not ($A = 0$). This framework allows us to define causal effects in terms of these unobservable potential outcomes.

To estimate causal effects, we rely on the following key assumptions:

Assumption 1 (Consistency) The observed response is consistent such that $Y(a) = Y \mid A = a$.

Assumption 2 (Positivity) The propensity score $\pi_a(W) := \mathbb{P}(A = a \mid W) \in (\epsilon, 1-\epsilon)$ for some $\epsilon \in (0, 1/2)$.

The positivity assumption is also known as the overlap condition or the common support condition. It requires that the two functions $\mathbb{P}(A = 1 \mid X = x)$ and $\mathbb{P}(A = 0 \mid X = x)$ share the same support in confounder values $X = x$ (corresponding to the overlap condition or common support condition). Violations of this assumption are typically diagnosed by inspecting whether estimated propensity scores are extremely close to 0 or 1, which suggests regions of the covariate space where treatment assignment is nearly deterministic.

Assumption 3 (No unmeasured confounders) $A \perp\!\!\!\perp Y(a) \mid W$, for all $a \in \{0,1\}$.

Under these assumptions (Assumptions 1–3), the observed outcome Y is conditionally independent of the treatment A, given the covariates W. This allows us to estimate the expected potential outcome for gene j under treatment ($a = 1$) or control ($a = 0$) as:

$$\mathbb{E}[Y_j(a)] = \psi_j(W, a) := \mathbb{E}[\mu_j(W, a)],$$

where $\mu_j(W, a) = \mathbb{E}[Y_j \mid W, A = a]$ is a regression function that models the relationship between covariates, treatment, and outcomes.

Suppose we have a dataset $\mathcal{D} = \{O_1, \ldots, O_n\}$ consisting of i.i.d. samples from the same distribution as O. Let $\mathbb{E}_n$ denote the empirical measure over $\mathcal{D}$, defined as:

$$\mathbb{E}_n[f(O)] = n^{-1} \sum_{i=1}^{n} f(O_i),$$

for any measurable function f. Equivalently, this computes the expectation with respect to the empirical distribution that puts weights $1/n$ on each observed data point. This represents the sample average of a function evaluated on all observations in the dataset.

A naive plug-in estimator for ψ_j can then be constructed by replacing the true regression function $\mu_j(W, a)$ with its estimated counterpart $\widehat{\mu}_j(W, a)$ and using sample averages to approximate expectations. The resulting estimator is:

$$\widehat{\psi}_j^{\mathrm{PI}} = \mathbb{E}_n[\widehat{\mu}_j(W, a)] = n^{-1} \sum_{i=1}^{n} \widehat{\mu}_j(W_i, a).$$

This plug-in estimator provides an estimate of the expected potential outcome by averaging predictions from the estimated regression model over all observations in the dataset.

While Assumptions 1–3 are foundational for causal inference, violations of the no unmeasured confounders assumption (Assumption 3) are common in real-world applications (5, 6). For instance, in single-cell transcriptomic studies, technical factors such as batch effects or biological heterogeneity (e.g., cell size or cell cycle stage) may act as unmeasured confounders. These unmeasured variables can bias estimates of causal effects by introducing spurious associations between treatment and outcome. Addressing this limitation motivates the need for methods that explicitly model and adjust for unmeasured confounders.

S2.2 Target estimands

For semiparametric inference, a target estimand is a distributional functional of the observed random variables. For example, we can consider the average treatment effects (ATE), the standardized average treatment effect (SATE), the average treatment effect in levels or fold change (FC), and the LFC. Below, we define these estimands:

- ATE: $\tau_j^{\mathrm{ATE}} = \mathbb{E}[Y_j(1) - Y_j(0)]$.
- SATE: $\tau_j^{\mathrm{SATE}} = \mathbb{E}[Y_j(1) - Y_j(0)] / \sqrt{\mathrm{Var}(Y_j(0))}$.
- ATE in levels: $\tau_j^{\mathrm{FC}} = \mathbb{E}[Y_j(1) - Y_j(0)] / \mathbb{E}[Y_j(0)]$.

- LFC: $\tau_j^{\mathrm{LFC}} = \log(\mathbb{E}[Y_j(1)]/\mathbb{E}[Y_j(0)])$.

Here, we use Y_j to denote the random variable of the jth outcome and $(Y_j(0), Y_j(1))$ to denote its potential outcomes. Next, we present the corresponding influence functions under the identification assumptions, Assumptions 1–3. Before we present the influence functions, we introduce the uncentered influence function for $\mathbb{E}[Y_j(a)]$ and $\mathbb{E}[Y_j(0)^2]$:

$$\phi_{ja}(O;\pi_a,\mu_{ja}) = \frac{\mathbb{1}\{A=a\}}{\pi_a(W)}(Y_j - \mu_{ja}(W)) + \mu_{ja}(W), \qquad a = 0,1$$
$$\phi_{j2}(O;\pi_0,\mu_{j2}) = \frac{\mathbb{1}\{A=0\}}{\pi_0(W)}(Y_j^2 - \mu_{j2}(W)) + \mu_{j2}(W),$$

where $\mu_{ja}(W) = \mathbb{E}[Y_j \mid W, A=a]$ for $a = 0,1$ and $\mu_{j2}(W) = \mathbb{E}[Y_j^2 \mid W, A=0]$. Note that the (centered) influence function of $\mathbb{E}[Y(a)]$ is given by $\phi_{ja}(O;\pi_a,\mu_{ja}) - \mathbb{E}[Y_j(a)]$. It follows that

$$\eta_j^{\mathrm{ATE}}(O;\pi,\mu_j) = \phi_{j1} - \phi_{j0} - \tau_j^{\mathrm{ATE}}.$$

The efficient centered influence function of τ_j^{SATE} is given by

$$\eta_j^{\mathrm{SATE}}(O;\pi,\mu_j) = \frac{\phi_{j1}-\phi_{j0}}{\sqrt{\mathbb{V}[Y_j(0)]}} - \tau_j^{\mathrm{SATE}}\left[\frac{\phi_{j2} + \mathbb{E}[Y_j(0)^2] - 2\mathbb{E}[Y_j(0)]\phi_{j0}}{2\mathbb{V}[Y_j(0)]}\right].$$

See for example, Equation (6) of (10) and Equation (4.3) of (7). Similarly, the efficient influence function of τ_j^{FC} is given by

$$\eta_j^{\mathrm{FC}}(O;\pi,\mu_j) = \frac{\phi_{j1}-\phi_{j0}}{\mathbb{E}[Y_j(0)]} - \frac{\tau_j^{\mathrm{FC}}\phi_{j0}}{\mathbb{E}[Y_j(0)]}$$
$$\eta_j^{\mathrm{LFC}}(O;\pi,\mu_j) = \frac{\phi_{j1}}{\mathbb{E}[Y_j(1)]} - \frac{\phi_{j0}}{\mathbb{E}[Y_j(0)]}.$$

In the current paper, we restrict our focus to LFC; however, our implementation also allows the computation and inference using other estimands listed above. When computing the LFCs, we use the size-normalized counts Y_{ij}/s_i adjusted by the size factors s_i in place of the raw count Y_{ij}. This is akin to taking a weighted average of the sample to estimate ATE (and, subsequently, LFC). Otherwise, the effect will be driven by cells with large library sizes.

CATE. Under standard identification assumptions of consistency, conditional exchangeability, and positivity as in Assumptions 1–3, the conditional average treatment effect (CATE) is identified by $\tau_j(w) = \mu_{j1}(w) - \mu_{j0}(w)$. This also applies to conditional log-fold change. When one is only interested in the conditional effects in a subset of variables $\mathcal{S} \subset [d_W + a]$, the DR-learner readily accommodates runtime confounding through the decomposition $\tau_{\mathcal{S}}(w) = \mathbb{E}[\phi(O) \mid W_{\mathcal{S}} = w_{\mathcal{S}}]$. This decomposition implies that one may estimate $\tau_{\mathcal{S}}(w)$ by regressing $\phi(O)$ on $W_{\mathcal{S}}$, i.e., modifying the final regression step of the DR-learner.

S2.3 False discovery rate control

Genomic studies often involve testing thousands of hypotheses simultaneously, making it crucial to control statistical Type I errors. Two widely recognized error rate metrics are the Family-Wise Error Rate (FWER) and the False Discovery Rate (FDR), each suited to different contexts. Consider p hypothesis tests, let $\mathcal{S} \subset \{1,\ldots,p\}$ denote the set of discoveries, and $\mathcal{H}_0 \subset \{1,\ldots,p\}$ denote the set of true null hypotheses. The false discovery proportion (FDP) is defined as the ratio of false positives to total discoveries:

$$\mathrm{FDP} = \frac{|\mathcal{S} \cap \mathcal{H}_0|}{|\mathcal{S}| \vee 1}.$$

The FWER controls the probability of making at least one false discovery:

$$\mathrm{FWER} := \mathbb{P}(\mathrm{FDP} > 0) \leq \alpha,$$

where $\alpha \in (0,1)$ is a predefined significance level. This stringent control is particularly useful in scenarios where even a single false positive is unacceptable. However, FWER control often leads to reduced statistical power, especially in high-dimensional settings with many hypotheses, potentially overlooking true effects.

In contrast, FDR control provides a more balanced approach by controlling the expected proportion of false discoveries among all discoveries:

$$\mathrm{FDR} := \mathbb{E}[\mathrm{FDP}] \leq \alpha.$$

Algorithm S2 Multiple testing on standardized treatment effects

Input: The estimated influence function values $\widehat{\eta}_{ij}$, the estimated variance $\widehat{\sigma}_j^2$ for $i = 1,\dots,n$ and $j = 1,\dots,p$. The FDP exceedance threshold c, the FDP exceedance probability α, and the number of bootstrap samples B. The threshold $\widetilde{c}$ to exclude genes with small variation.

Initialize the iteration number $\ell = 1$, the candidate set $\mathcal{A}_1 = \{j \in [p] \mid \widehat{\sigma}_j^2 \geq \widetilde{c}\}$, the set of discoveries $\mathcal{V}_1 = \varnothing$, and the maximal statistic of $M_1 = \max_{j\in\mathcal{A}_1} |t_j|$.

while not converge **do**

  Let $\boldsymbol{D}_{n\ell} = \mathrm{diag}((\widehat{\sigma}_j)_{j\in\mathcal{A}_\ell})$ be the diagonal matrix of the estimated standard deviations and $\widehat{\boldsymbol{\eta}}_{i\ell} = (\widehat{\eta}_{ij})_{j\in\mathcal{A}_\ell}$ be the vector of estimated influence function values at iteration ℓ.

  Draw multiplier bootstrap samples $\boldsymbol{g}_\ell^{(b)} = (\sqrt{n}\boldsymbol{D}_{n\ell})^{-1}\sum_{i=1}^n \varepsilon_{i\ell}^{(b)} \widehat{\boldsymbol{\eta}}_{i\ell}$, where $\varepsilon_{i\ell}^{(b)}$'s are independent samples from $\mathcal{N}(0,1)$ for $i = 1,\dots,n$ and $b = 1,\dots,B$.

  Compute the maximal statistic $M_\ell = \max_{j\in\mathcal{A}_\ell} |t_j|$.

  Estimate the upper α-quantile of M_ℓ under $H_0^{(\ell)} : \tau_j^* = 0,\ \forall\, j \in \mathcal{A}_\ell$ by

$$\widehat{q}_\ell(\alpha) = \inf\left\{ x \,\middle|\, \frac{1}{B}\sum_{b=1}^{B} \mathbb{1}\{\|\boldsymbol{g}_\ell^{(b)}\|_\infty \leq x\} \geq 1-\alpha \right\}$$

  Set $j_\ell = \mathrm{argmax}_{j\in\mathcal{A}_\ell} |t_j|$ and $\mathcal{A}_{\ell+1} = \mathcal{A}_\ell \setminus \{j_\ell\}$.

  if $M_\ell > \widehat{q}_\ell(\alpha)$ **then**

    Set $\mathcal{V}_{\ell+1} = \mathcal{V}_\ell \cup \{j_\ell\}$.

  else

    Declare the standardized treatment effects in $\mathcal{A}_\ell$ are not significant stop the step-down process.

  end if

  $\ell \leftarrow \ell + 1$.

end while

Augmentation: Set $\mathcal{V}$ to be the union of $\mathcal{V}_\ell$ and the $\lfloor |\mathcal{V}_\ell| \cdot c/(1-c) \rfloor$ elements from $\mathcal{A}_\ell$ with largest magnitudes of t_j.

Output: The set of discoveries $\mathcal{V}$.

This approach enhances power in multiple testing scenarios and has become the standard for differential expression analysis in genomics due to its ability to identify more significant features while maintaining a low proportion of false positives (1). Importantly, FDR controls the *expected* proportion of false discoveries across repeated experiments but does not guarantee bounds on FDP in any single experiment. This distinction becomes critical in genomic studies where test statistics are often highly dependent, leading to variability in FDP across experiments.

To address limitations of standard FDR procedures, such as their inability to capture FDP variability in a single experiment, alternative error control metrics like False Discovery Exceedance (FDX) have been proposed:

$$\mathrm{FDX} := \mathbb{P}(\mathrm{FDP} \geq c) \leq \alpha,$$

for a threshold $c \in (0,1)$. FDX provides stricter control by limiting the probability that FDP exceeds a predefined threshold c. This makes it particularly useful in applications where minimizing false positives is critical or when restricting analysis to a small subset of discoveries is desired.

To ensure robust error rate control tailored to genomic applications, causarray implements two complementary strategies for FDR control: (i) Benjamini–Hochberg (BH) Procedure: The BH procedure (1) is applied directly to P-values obtained from the semiparametric estimation framework. BH controls the FDR under independence or specific positive dependence structures among test statistics. (ii) Gaussian Multiplier Bootstrap: For tighter control of FDP variability, when test statistics are highly dependent, causarray incorporates a Gaussian multiplier bootstrap approach (Algorithm S2). It simulates null distributions to estimate FDP more accurately and provides robust FDR control even under complex dependence structures (7).

The choice between the BH and the Gaussian multiplier bootstrap depends on the dependency structure among test statistics. While BH is computationally efficient and widely used, it may not adequately control FDR under strong dependencies. The Gaussian multiplier bootstrap, on the other hand, accounts for complex dependency structures and provides more accurate bounds on FDP variability. Additionally, incorporating FDX offers an extra layer of conservatism for applications where minimizing false positives is critical. By offering these complementary strategies, causarray ensures robust error rate control tailored to diverse genomic applications while balancing power and error control.

Supplementary Information S3: Data simulation and analysis

S3.1 Data simulation

We consider two simulation settings. In the first simulation, we generate cells from zero-inflated Poisson distributions. In the second simulation, we use a specialized single-cell simulator Splatter (27) to generate cells with batch effects. Both simulations include 1 observed covariate and 4 unmeasured confounders. The details of the simulation are provided below.

Pseudo-bulk expression simulation details. The pseudo-bulk expression data are generated using a Poisson distribution with a zero-inflation component. The setup involves generating a latent signal matrix influenced by random noise and specific parameters. The data generation process is described in Algorithm S3 in detail. For experimental results in Fig. 2, we set $d = 2$ and $r^* = 1$, and vary $n \in \{100, 500, 1000, 5000\}$. For causarray, RUV, and RUV-III-NB, we provide the number of latent factors in $r \in \{2, 4, 6\}$. Because the simulated data consists of 3 cell types, which may be explained with 3 additional degrees of freedom, the best possible choice of the number of latent factors would be $r = 4$. The metrics for each experimental setting are calculated using 50 simulated datasets with varying random seeds.

Algorithm S3 Data generation process for pseudo-bulk gene expressions.

Input: Number of subjects n, number of covariates d, number of latent factors r_0, number of cells per subject $m = 10$, number of genes $p = 2000$, number of significant genes $s = 100$, and zero-inflation probability $\psi = 0.1$.

1: (Signals) The p-dimensional signal is derived from multiplying the signal strength by a Beta distributed vector, modified by a random sign flip:

$$\beta_j \sim 0.5 \times \text{Beta}(1, 0.1) \times (2 \times \text{Bernoulli}(0.5) - 1), \qquad j = 1, \ldots, s,$$

and $\beta_j \equiv 0$ for $j = s+1, \ldots, p$.

2: (Cell types) The 3 cell types are generated with means $\{-0.5, 0, 0.5\}$ and standard deviations drawn from $\text{Uniform}(0.5, 1)$. For n subjects, the cell type assignment is randomly sampled from $\text{Categorical}(3)$ and the cell-type specific means and scales are stored as n-dimensional vectors $\boldsymbol{\mu}_{\text{ct}}$ and $\boldsymbol{\sigma}_{\text{ct}}$.

3: (Covariates) Sample d observed covariates $\boldsymbol{W}_{\cdot j} \sim 0.5\boldsymbol{\sigma}_{\text{ct}} \times \mathcal{N}_n(\boldsymbol{\mu}_{\text{ct}}, \mathbf{1}_n)$ for $j = 1, \ldots, d$, and unobserved covariates $\boldsymbol{W}_{\cdot j} \sim 0.5\boldsymbol{\sigma}_{\text{ct}} \times \mathcal{N}_n(\boldsymbol{\mu}_{\text{ct}}, \mathbf{1}_n)$ for $j = d+1, \ldots, d+r_0$.

4: (Treatments) Sample $\boldsymbol{A} \sim \text{Bernoulli}(\text{Logistic}(\boldsymbol{W}\boldsymbol{\alpha}))$ where $\boldsymbol{\alpha} \sim \mathcal{N}_d(\mathbf{0}_{d+r_0}, (4(d+r_0))^{-1/2}\mathbf{1}_{d+r_0})$.

5: (Coefficient matrix) Sample $b_{0j} \sim \text{Beta}(2, 1)$ and $\boldsymbol{B}_{\cdot j} \sim \mathcal{N}_d(\mathbf{0}_{d+r_0}, (4(d+r_0))^{-1/2}\mathbf{1}_{d+r_0})$ for $j = 1, \ldots, p$.

6: (Natural parameters) Let $\boldsymbol{\Theta} = \mathbf{1}\boldsymbol{b}_0^\top + \boldsymbol{W}\boldsymbol{B}^\top + \boldsymbol{A}\boldsymbol{\beta}^\top$.

7: (Single-cell observations) Let $\boldsymbol{Y}^{\text{sc}} \in \mathbb{R}^{n \times p \times m}$ with $\boldsymbol{Y}^{\text{sc}}_{\cdot\cdot\ell} \sim \text{Bernoulli}((1-\psi) \times \mathbf{1}_{n \times p}) \times \text{Poisson}(\exp(\boldsymbol{\Theta}))$ for $\ell = 1, \ldots, m$.

8: (Bulk observations) Let $\boldsymbol{Y} \in \mathbb{R}^{n \times p}$ with $\boldsymbol{Y} = \sum_{\ell=1}^{m} \boldsymbol{Y}^{\text{sc}}_{\cdot\cdot\ell}$.

Output: Covariates $\boldsymbol{W}$, treatment $\boldsymbol{A}$, single-cell gene expression $\boldsymbol{Y}^{\text{sc}}$, and pseudo-bulk gene expression $\boldsymbol{Y}$.

Single-cell expression simulation details. The single-cell expression data are generated by Splatter (27). Splatter explicitly models the hierarchical Gamma-Poisson processes that give rise to data observed in scRNA-seq experiments and can model the multiple-faceted variability. The data is generated from the `splatSimulate` function from the Splatter (1.26.0) package (27). When calling this function, the treatment effects are simulated with the parameters: `group.prob = c(0.5, 0.5), method = "groups", de.prob=0.05,de.facLoc=1., de.facScale=0.5, de.downProb=0.5`; the dropout effects are simulated with the parameters: `dropout.type="experiment", dropout.mid=20, dropout .shape=0.001`; the batch effects are simulated with the parameters: `batch.facLoc=noise, batch.facScale=0.5`; while all the other parameters are the same as returned by the function `newSplatParams`. For experimental results in Fig. S2, we generate $d = 1$ covariates and $r = 4$ unmeasured confounders. We first generate $(d+r+1)/2$ batches with equal sample sizes, which account for $d+r$ degrees of freedom. To simulate varying confounding levels, we set `noise` in $\{0.1, 0.2, 0.3\}$. The metrics for each experimental setting are calculated using 50 simulated datasets with varying random seeds.

S3.2 Benchmarking methods

To evaluate the performance of differential expression (DE) testing, we compare causarray with several established methods, both with and without confounder adjustment. Several linear methods, such as SVA (12), CATE (25), BConf (18), and dSVA (11), have been developed for confounder adjustment in bulk transcriptomic or DNA methylation data. Among these, CATE has demonstrated superior FDR control under linear models. However, such methods are less suitable for single-cell RNA-seq data due to sparsity and nonlinear mean–variance relationships. GCATE (6), based on a generalized linear model (GLM), outperformed CATE on both single-cell and pseudo-bulk data. As CATE, BConf, and dSVA perform similarly (19), we focus on methods better suited for single-cell data in this study.

Many methods aim to estimate confounding while performing single-cell treatment effect estimation, including Mixscape, contrastiveVI, cellOT, CPA, and PS (2, 14, 20, 24, 26). We compare causarray against CINEMA-OT and Mixscape as representatives of these approaches (optimal transport and matching).

Comparison methods included in simulations are grouped into two categories based on whether they account for unmeasured confounders.

Methods without confounder adjustment include:

- Wilcoxon rank-sum test: This nonparametric test is applied to deviance residuals obtained by regressing gene expression counts on measured covariates using a negative binomial GLM. The deviance residuals serve as input for the test, which does not explicitly account for unmeasured confounders.
- DESeq2 (15): This widely used method fits a negative binomial GLM to gene expression counts and adjusts for measured covariates. However, it does not account for unmeasured confounders, which may bias results in the presence of hidden variation.

Methods with confounder adjustment include:

- CoCoA-diff (R package `mmutilR 1.0.5`) (21): Designed for individual-level case-control studies, CoCoA-diff prioritizes disease genes by adjusting for confounders estimated from parametric models. We set `knn = 50` for cell matching, as suggested in the original publication, while keeping the remaining parameters unchanged. After adjusting for these confounders, the Wilcoxon rank-sum test is applied to the adjusted residuals, as recommended in the original paper.
- CINEMA-OT and CINEMA-OT-W (Python package `cinemaot 0.0.3`) (4): CINEMA-OT separates confounding sources of variation from perturbation effects using optimal transport matching to estimate counterfactual cell pairs. Compared to CINEMA-OT, CINEMA-OT-W further adjusts for differences in propensity scores between treated and control cells by matching before independent component analysis. We applied CINEMA-OT to library-size-normalized and log1p-transformed counts, setting the smoothness parameter to 1e-3 while maintaining all other parameters at their default values. Similar to CoCoA-diff, the Wilcoxon rank-sum test is applied to the adjusted residuals of CINEMA-OT or CINEMA-OT-W.
- Mixscape (20), a popular method based on matching for modeling perturbation effects. We applied the implementation from (4) to library-size-normalized and log1p-transformed counts.
- RUV-III-NB (R package `ruvIIInb 0.8.2.0`) (23): This method normalizes gene expression data using pseudo-replicates and a negative binomial model to remove unwanted variation induced by library size differences. The Kruskal-Wallis test (equivalent to the Wilcoxon test for two-group comparisons) is then applied to log-percentile adjusted counts, as suggested by the authors. However, RUV-III-NB does not directly adjust for library size and its ability to control FDR remains unclear, as it was not demonstrated in their experiments.
- RUV (R package `ruv 0.9.7.1`) (22): RUVr is used to estimate unmeasured confounders, which are then incorporated into DESeq2 for statistical inference based on both observed and estimated covariates. Before running RUV, we successively use the functions `calcNormFactors`, `estimateGLMCommonDisp`, `estimateGLMTagwiseDisp`, and `glmFit` of edgeR package (4.0.16) (3) to extract residuals not explained by observed covariates and treatments.

This comprehensive benchmarking enables a thorough evaluation of each method's ability to address unmeasured confounder estimation and perform robust statistical inference in simulated data settings. Users interested in applying these methods to new datasets may consider additional hyperparameter tuning to potentially improve performance. However, this typically requires defining a task-specific objective function and performing cross-validation. These steps are not straightforward in real-world applications where the true set of differentially expressed genes is unknown.

S3.3 Evaluation metrics

To compare the performance of different methods, we use four evaluation metrics, focusing on two aspects: confounder estimation and biological signal preservation. DESeq2 and Wilcoxon are excluded from confounder estimation evaluation as they do not estimate unmeasured confounders or counterfactuals.

The performance of confounder estimation is assessed using two clustering-based metrics: Adjusted Rand Index (ARI) and Average Silhouette Width (ASW) (16). These metrics evaluate the quality of mixing in response and confounder spaces, respectively. Formally, measures the similarity between the clustering results based on the estimated control responses $Y(0)$ and the true cell-type labels of the same samples. It adjusts for similarities that occur by chance:

$$\mathrm{ARI} = \frac{\sum_{ij}\binom{n_{ij}}{2} - [\sum_i \binom{a_i}{2}\sum_j \binom{b_j}{2}]/\binom{n}{2}}{\frac{1}{2}[\sum_i \binom{a_i}{2} + \sum_j \binom{b_j}{2}] - [\sum_i \binom{a_i}{2}\sum_j \binom{b_j}{2}]/\binom{n}{2}},$$

where n is the total number of samples, n_{ij} is the number of samples in both cluster i and partition j, a_i is the sum over rows in the contingency table, and b_j is the sum over columns. Higher ARI values indicate better conservation of cell identity based on estimated counterfactuals compared to true labels. When cell type is not the dominant confounder, as in our pseudo-bulk expression simulation, a larger ARI reflects better performance. In contrast, when cell type is the dominant confounder, as in the single-cell simulation, we seek to erase that signal, so a smaller ARI indicates more effective deconfounding. ARI ranges from -1 (complete disagreement) to 1 (perfect agreement), with 0 indicating random clustering. On the other hand, ASW quantifies how well each sample fits within its assigned cluster compared to other clusters. It is defined as:

$$\mathrm{ASW} = \frac{1}{n}\sum_{i=1}^{n}\frac{b(i)-a(i)}{\max\{a(i),b(i)\}},$$

where $a(i)$ is the average dissimilarity of sample i to all other samples within its cluster, and $b(i)$ is the average dissimilarity to samples in the nearest neighboring cluster. ASW values range from -1 to 1, with higher values indicating better-defined clusters (16). For both metrics, median scores are scaled between 0 and 1 across methods within each simulation setup. For these two metrics, we use the implementations from the `scib` (`1.1.5`) package (16).

To evaluate biological signal preservation, we use False Positive Rate (FPR) and True Positive Rate (TPR), which are standard metrics derived from confusion matrices: PR quantifies the proportion of false positives among all true negatives:

$$\mathrm{FPR} = \frac{\mathrm{FP}}{\mathrm{FP}+\mathrm{TN}},$$

where FP and TN are false positives and true negatives, respectively. A lower FPR indicates fewer false discoveries relative to true negatives. Also known as sensitivity or recall, TPR measures the proportion of true positives among all actual positives:

$$\mathrm{TPR} = \frac{\mathrm{TP}}{\mathrm{TP}+\mathrm{FN}},$$

where TP and FN are true positives and false negatives, respectively. A higher TPR indicates better detection of true signals. These metrics provide complementary insights: FPR evaluates specificity by penalizing false discoveries, while TPR assesses sensitivity by rewarding correct detections. Together, they measure how well a method balances identifying true signals while avoiding false discoveries.

For the benchmarking methods presented in the paper, by design, the data are appropriate for either bulk or single-cell analysis, but not both. When many subjects have been measured, there is an intra-subject correlation among the cells. Analyzing at the pseudo bulk level circumvents this issue by treating subjects as the experimental unit. When the data is derived from a cell line or biological clones, the correlation between cells is negligible, and it is acceptable to use cells as the experimental unit. In this setting, a pseudo bulk analysis would not be advised. We describe the two simulation setups below.

S3.4 Single-cell Perturb-Seq dataset

We utilize the Perturb-Seq dataset from (9), which enables high-resolution transcriptomic profiling of genetic perturbations in excitatory neurons. This scalable platform systematically investigates gene functions across diverse cell types and perturbation conditions, providing critical insights into neurodevelopmental processes (9). We focus on excitatory neurons of the dataset, a key population implicated in neurodevelopmental disorders such as autism spectrum disorders and neurodevelopmental delay, with perturbations targeting genes involved in neuronal development and synaptic function (9).

For preprocessing, if a given perturbation appears in fewer than 50 cells, all cells under that condition are excluded from the analysis. We then filter out genes expressed in fewer than 50 cells, resulting in a dataset containing 2926 cells under 30 perturbation conditions. The GFP (Green Fluorescent Protein) condition is used as a negative control to benchmark the effects of other perturbations by providing a baseline for comparison in downstream analyses. After filtering lowly expressed genes with a maximum count of fewer than 10, we retain 3221 genes.

The batch design is highly correlated with perturbation conditions; therefore, it is not included as a covariate in the model for testing. Instead, only the intercept is included as a covariate. For propensity score estimation, we incorporate the logarithm of library sizes as an additional covariate to account for technical variability and use GLM as the propensity score model. We performed a focused *Satb2* analysis to identify a biologically meaningful signal detected by causarray but missed by other correction methods, and then linked that signal to latent confounding. For *Satb2*, causarray identifies GO:0021953 (central nervous system neuron differentiation) with adjusted p-value 1.97e-05, while this term is absent in the RUV *Satb2* GO list. We then examined the genes underlying this GO term. The term contains 34 genes, including 26 genes classified as causarray-only vs RUV using the pre-specified criterion (causarray padj < 0.1, RUV padj $>= 0.1$ or missing), which represents biologically coherent genes preferentially identified by causarray (Fig. S5c). To characterize the confounder(s), we used the causarray latent factors ($\widehat{U}$) and computed their correlation with all genes, and the causarray-only set of DE genes shows substantial association with latent factors (Fig. S5d), consistent with the interpretation that these genes are influenced by unmeasured variation not

explicitly modeled by standard alternatives. As one concrete example, *Hsp90ab1* in this GO term is strongly associated with latent confounding (maximum correlation = 0.419, best factor V7). Overall, these results support that causarray recovers biologically relevant Satb2 neurodevelopmental signals while explicitly accounting for latent confounding that can obscure or distort detection in other pipelines.

S3.5 Single-nucleus Alzheimer's disease dataset

This study integrates data from three single-nucleus RNA sequencing (snRNA-seq) datasets to investigate Alzheimer's disease (AD): the ROSMAP-AD dataset (17) and two datasets from the Seattle Alzheimer's Disease Brain Cell Atlas (SEA-AD) consortium (8), covering the middle temporal gyrus (MTG) and prefrontal cortex (PFC). These datasets provide complementary insights into AD pathology across different brain regions and donor cohorts.

The ROSMAP-AD dataset is derived from a single-nucleus transcriptomic atlas of the aged human prefrontal cortex, including 2.3 million cells from postmortem brain samples of 427 individuals with varying degrees of AD pathology and cognitive impairment (17). To ensure a balanced representation across subjects, we perform stratified down-sampling of 300 cells per subject, focusing on excitatory neurons while excluding two rare subtypes ('Exc RELN CHD7' and 'Exc NRGN'). This preprocessing results in a dataset with 124997 cells and 33538 genes.

Next, we create pseudo-bulk gene expression profiles by aggregating gene expression counts across cells for each subject. Genes expressed in fewer than 10 subjects are filtered out, resulting in a final dataset of 427 samples and 26,106 genes. Binary treatment is defined based on the variable
'age_first_ad_dx', which approximates the "age at the time of onset of Alzheimer's dementia." Covariates included in the analysis are 'msex' (biological sex), 'pmi' (postmortem interval), and 'age_death' (age at death). Missing values for 'pmi' are imputed using the median of observed values.

The SEA-AD data are obtained from a multimodal cell atlas of AD developed by the Seattle Alzheimer's Disease Brain Cell Atlas (SEA-AD) consortium (8). This resource includes snRNA-seq datasets from two brain regions: the middle temporal gyrus (MTG) and prefrontal cortex (PFC), covering 84 donors with varying AD pathologies.

For both MTG and PFC datasets, we perform stratified down-sampling of 300 cells per subject, focusing on excitatory neurons. Pseudo-bulk gene expression profiles are created by aggregating counts across cells for each subject. Genes expressed in fewer than 40 subjects are filtered out, resulting in final datasets with 80 samples and 24,621 genes for MTG and 80 samples and 25,361 genes for PFC. Covariates included in the analysis are 'sex', 'pmi', and 'Age_at_death'. These variables account for biological and technical variability across donors.

To enable comparative analyses across the three datasets (ROSMAP-AD, SEA-AD MTG, and SEA-AD PFC), we restrict the analysis to 15586 common genes that are expressed in all three datasets. Genes with a maximum expression count below 10 among subjects are excluded to ensure robust comparisons.

Functional analysis. We further compare functional enrichment results between causarray and RUV using gene ontology (GO) terms associated with DE genes. Both methods identify overlapping top functional categories related to key biological processes associated with AD pathology (Fig. S6c). Both methods detect GO terms relevant to neuronal development and synaptic functions, which are critical for understanding AD pathology. However, causarray shows distinct enrichment in categories such as "positive regulation of cell development" and "negative regulation of cell cycle', reflecting its increased sensitivity to synaptic and neurotransmission-related processes. In contrast, RUV's results exhibit more dataset-specific enrichments, such as biosynthetic processes in SEA-AD (PFC), apoptotic processes in SEA-AD (MTG), and catabolic processes in ROSMAP-AD (Fig. S6c). These findings suggest that causarray captures more generalizable biological signals across datasets. The visualization of the discovered networks, as defined as the top 5 GO terms and associated genes included in the top 100 DE gene discoveries, further highlights the enhanced sensitivity and comprehensiveness of causarray. Specifically, the causarray network contains 17 gene nodes and 81 edges, compared to 14 gene nodes and 57 edges in the RUV network (Fig. 4d). This greater interconnectedness in the larger causarray network suggests a more intricate and informative representation of underlying biological relationships, emphasizing its ability to capture broader and more relevant genetic factors associated with AD pathology.

Supplementary Information S4: Extra results

S4.1 Simulation

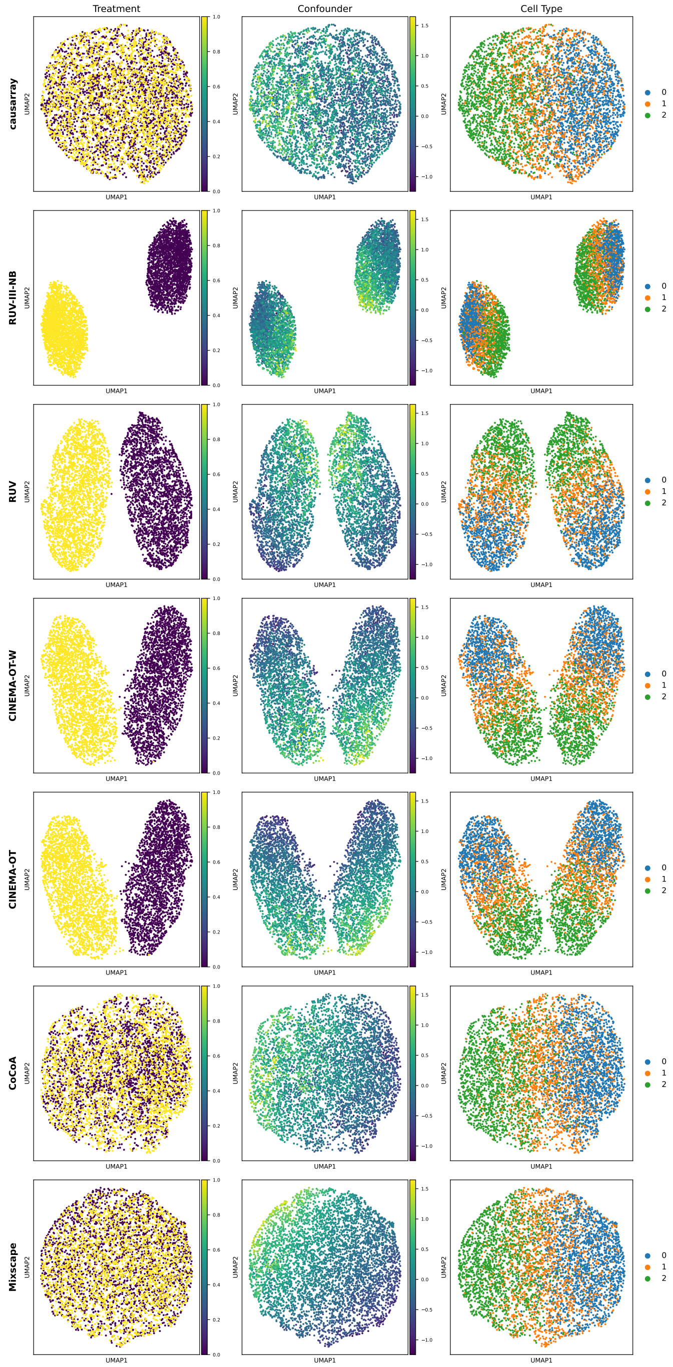

Fig. S1. UMAP visualization of different methods in the confounder space on synthetic pseudo-bulk expression data with $n = 5000$ observations. The three columns are colored by treatment assignment (yellow for treated and purple for control), confounder value, and cell type. Because treatment is only probabilistically linked to the hidden confounder, treated and control cells should overlap in the actual confounder space. An estimator that recovers this geometry will therefore display intermingled colors in the UMAP, whereas clear color separation signals residual confounding or broken overlap. An ideal representation should eliminate treatment structure (mixing) while preserving both the continuous confounder gradient and the cell-type clusters. The visualisations show such a pattern for causarray, and CoCoA-diff. In contrast, other methods retain two clearly separated treatment clouds, probably due to the nonlinearity and sparsity nature of count data. This also explains the inflated false discovery rates of some of the other methods.

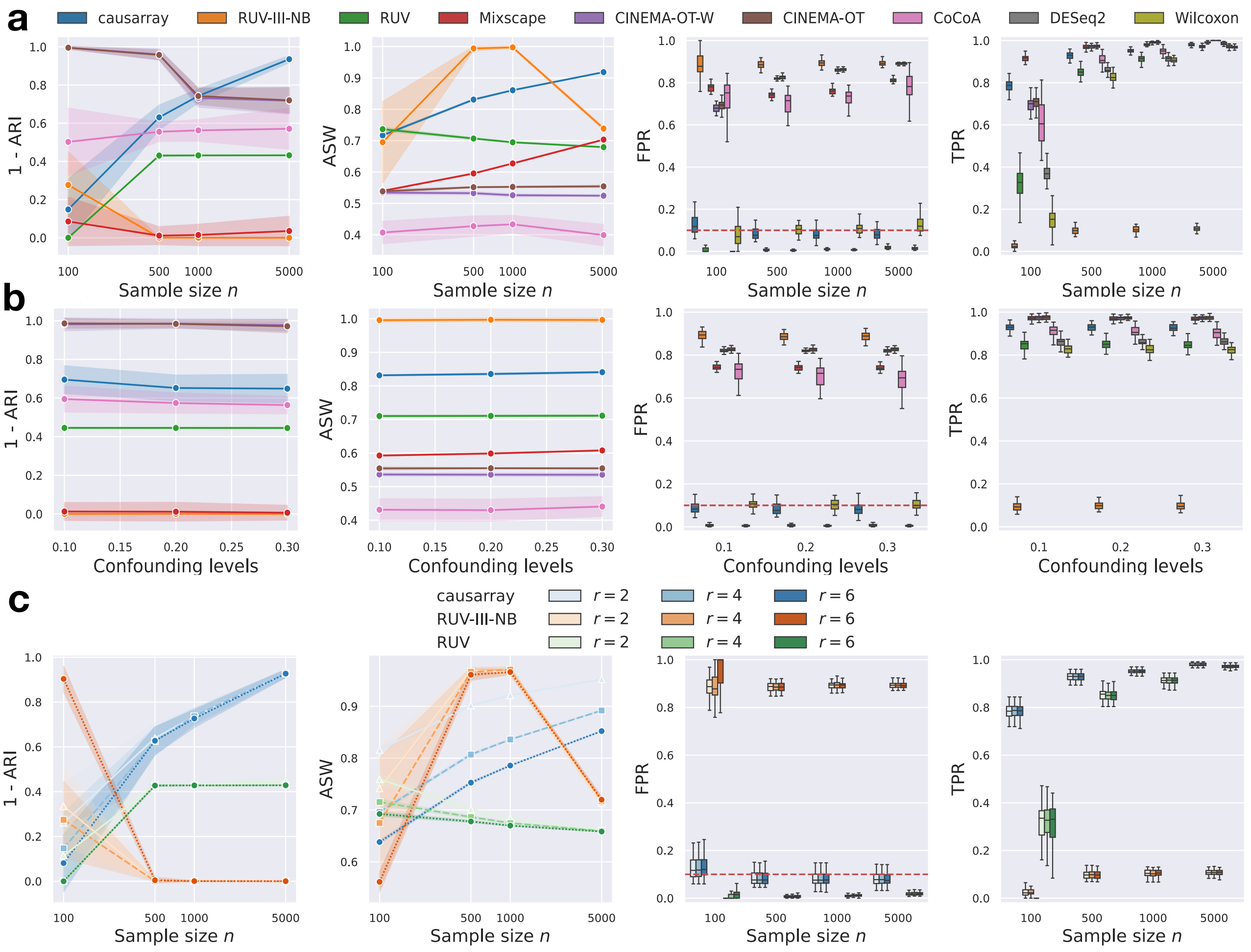

Fig. S2. Benchmarking of causarray against other methods for single-cell differential expression testing on synthetic single-cell expression data under unmeasured confounders. The metrics for each experimental setting are calculated using 50 simulated datasets with varying random seeds. **a,** Line plots and box plots of different validation metrics for causarray and other methods with $r = 4$ latent factors and a moderate confounding level. Line plots show mean ARI and ASW scores for confounder estimation (shaded region represents values within one standard deviation). Because cell type is the dominant confounder, a smaller ARI indicates more effective deconfounding. Box plots (FPR, false positive rate, and TPR, true positive rate) indicate the performance of biological signal preservation. For box plots, the median is used as the center, the top and bottom hinges represent the top and bottom quartiles, and whiskers extend from the hinge to the largest or smallest value no further than 1.5 times the interquartile range from the hinge. **b,** Line plots and box plots of different validation metrics for causarray and other methods with varying confounding effects with $n = 500$ samples). **c,** Line plots and box plots of different validation metrics for RUV, RUV-III-NB, and causarray, with varying numbers of latent factors.

S4.2 Perturb-seq data

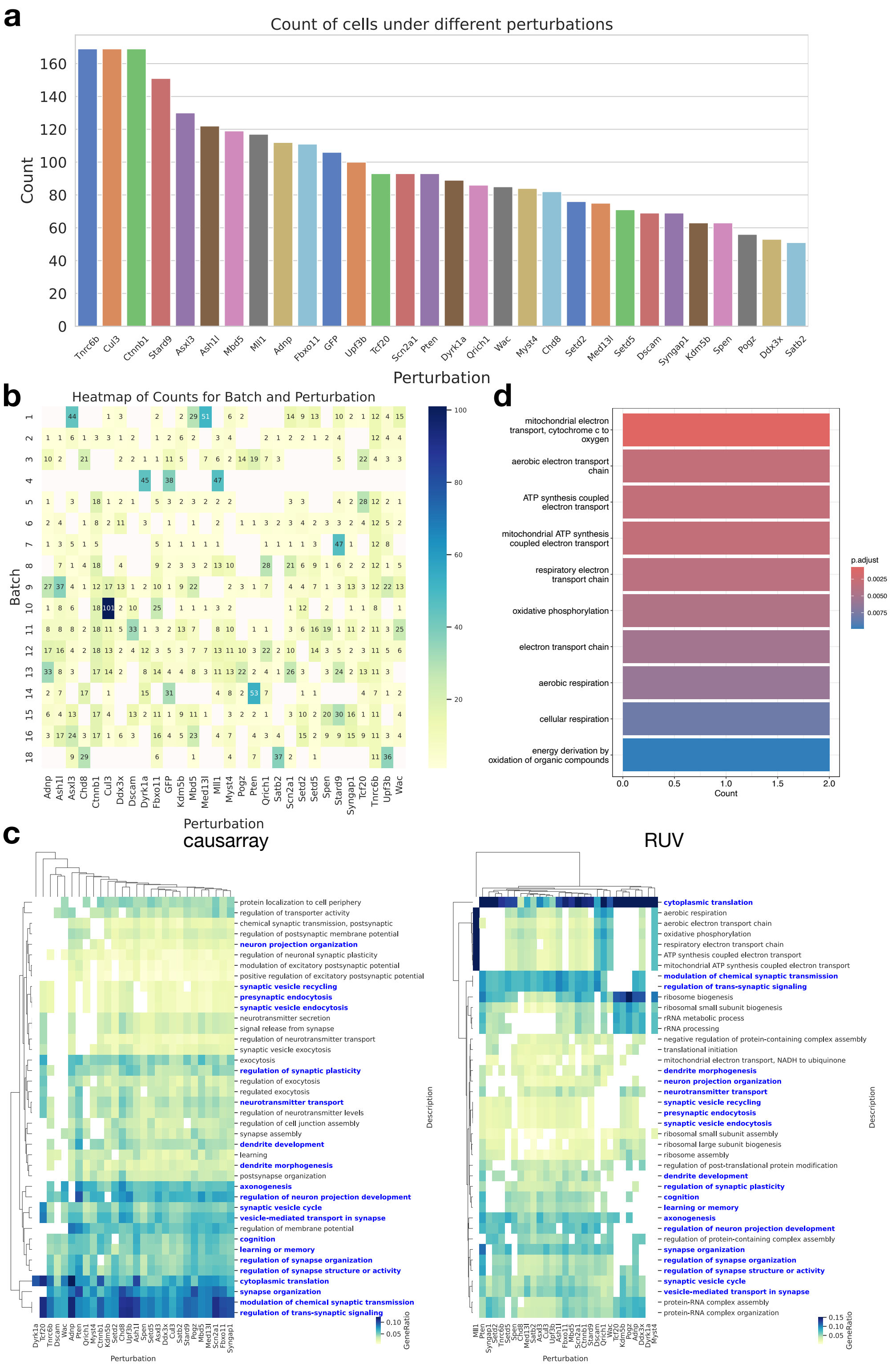

Fig. S3. Additional results on the Perturb-seq dataset. a, Barplot of the number of cells in each perturbation. **b,** Heatmap of the number of cells in each batch and perturbation. The batch design and the perturbation assignment of the Perturb-seq dataset are highly correlated. **c,** Clustermaps of GO terms enriched in discoveries (FDR<0.1) from causarray and RUV, respectively, where the common GO terms are highlighted in blue. Only the top 40 GO terms that have the most occurrences in all perturbations are displayed. **d,** Barplot of GO terms enriched in discoveries under *Mll1* perturbation from RUV.

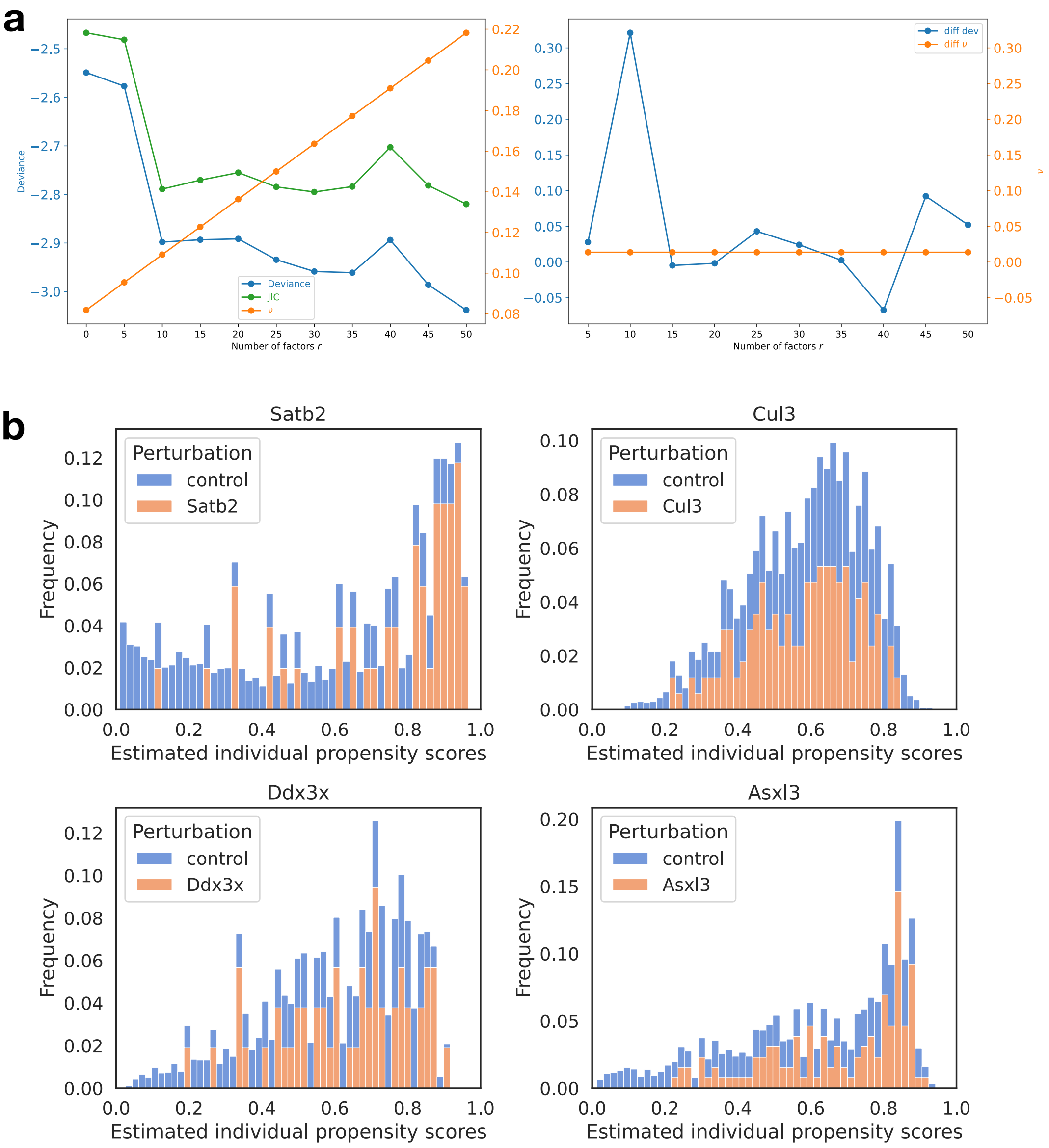

Fig. S4. Estimation results of causarray on the Perturb-seq dataset. **a,** The JIC criteria suggest the number of latent factors $r = 10$. **b,** Histograms of estimated propensity score for the top 4 perturbations ($Satb2$, $Cul3$, $Ddx3x$, and $Asxl3$) with most significant genes (adjusted P value < 0.1).

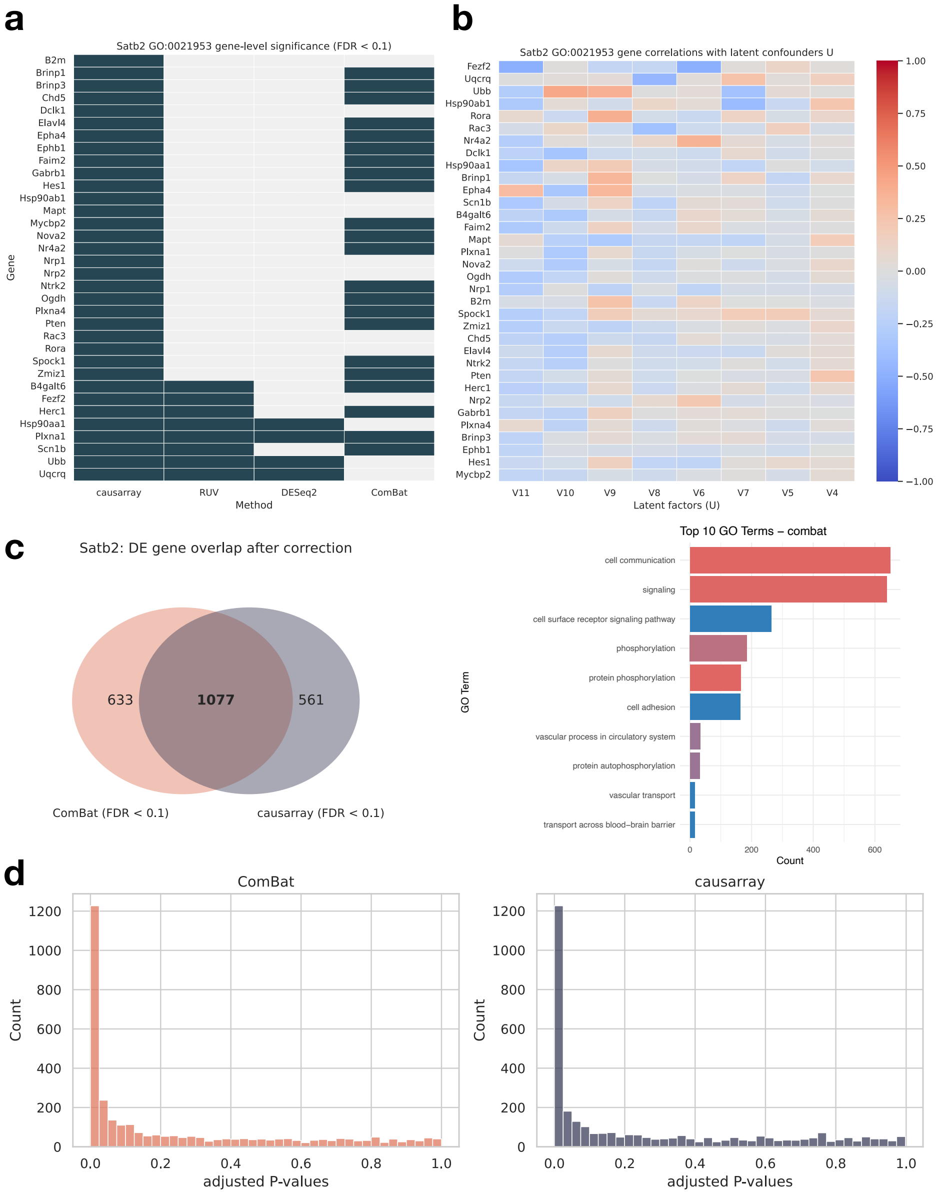

Fig. S5. Extra results for *Satb2* perturbation on the Perturb-seq dataset. a, The DE genes by different methods that are associated with GO:0021953. **c,** The heatmap of correlation between the estimated latent factors by causarray and distinct DE genes by causarray versus RUV. **c,** The numbers of DE genes by ComBat and causarray, and the top 10 GO terms associated with ComBat's DE genes. **d,** The distribution of adjusted P-values by ComBat and causarray.

S4.3 Alzheimer's data

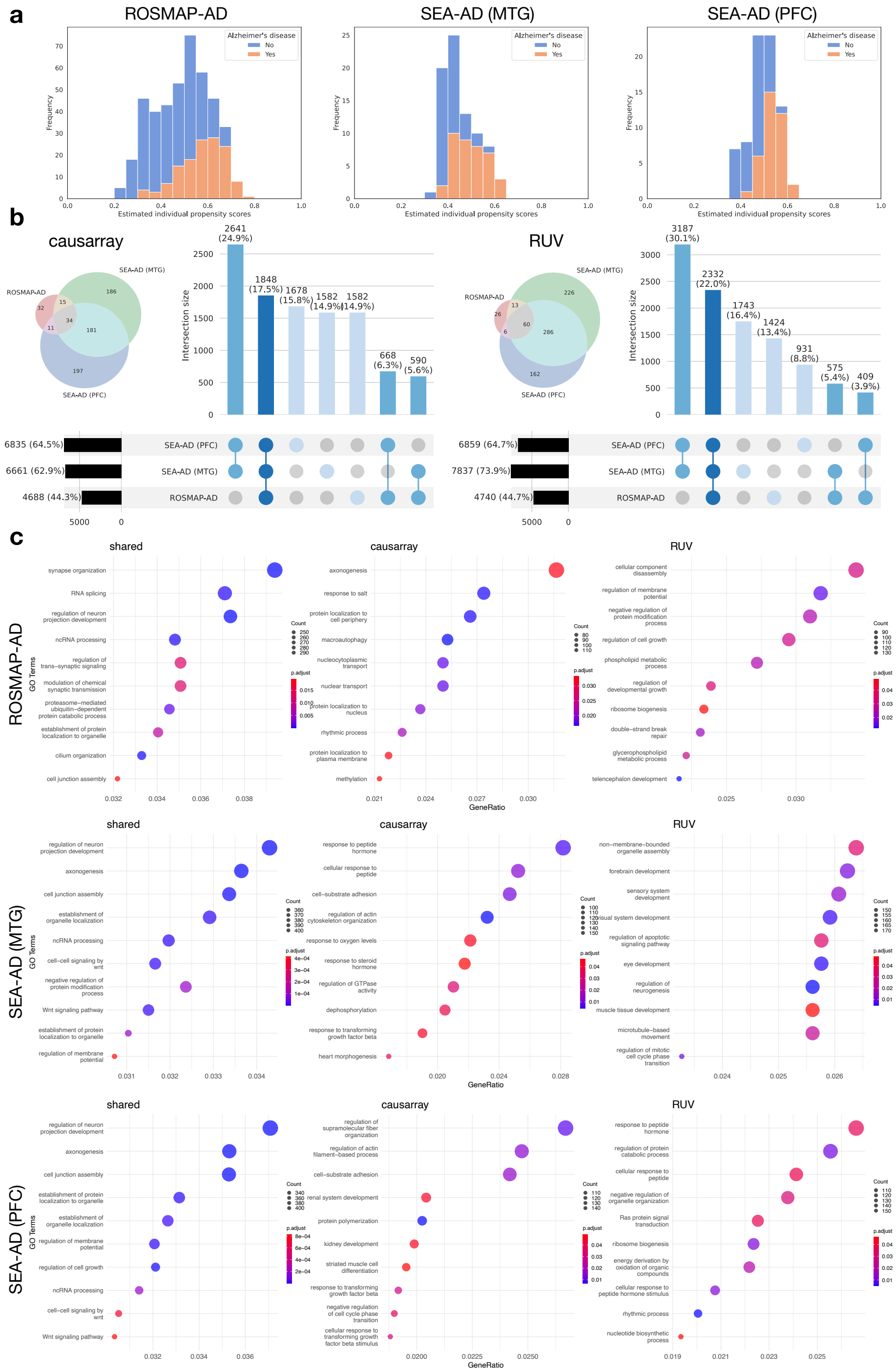

Fig. S6. Extra experimental results in AD datasets. a, Histogram of estimated propensity score in three AD datasets. Note that the estimated scores remain within a moderate range away from 0 and 1, and the propensity score distributions overlap well, indicating no violation of Assumption 2. **b,** DE genes by causarray and RUV over 15586 genes (adjusted P value < 0.1). Venn diagrams show the associated GO terms (adjusted P value < 0.05, $q < 0.2$). **c,** Top gene ontology terms of the shared and distinct discoveries by causarray and RUV.

1. Yoav Benjamini and Yosef Hochberg. Controlling the false discovery rate: a practical and powerful approach to multiple testing. *Journal of the Royal statistical society: series B (Methodological)*, 57(1):289–300, 1995.
2. Charlotte Bunne, Stefan G Stark, Gabriele Gut, Jacobo Sarabia Del Castillo, Mitch Levesque, Kjong-Van Lehmann, Lucas Pelkmans, Andreas Krause, and Gunnar Rätsch. Learning single-cell perturbation responses using neural optimal transport. *Nature methods*, 20(11):1759–1768, 2023.
3. Yunshun Chen, Lizhong Chen, Aaron TL Lun, Pedro L Baldoni, and Gordon K Smyth. edger 4.0: powerful differential analysis of sequencing data with expanded functionality and improved support for small counts and larger datasets. *bioRxiv*, pages 2024–01, 2024.
4. Mingze Dong, Bao Wang, Jessica Wei, Antonio H de O. Fonseca, Curtis J Perry, Alexander Frey, Feriel Ouerghi, Ellen F Foxman, Jeffrey J Ishizuka, Rahul M Dhodapkar, et al. Causal identification of single-cell experimental perturbation effects with cinema-ot. *Nature Methods*, pages 1–11, 2023.
5. Jin-Hong Du, Kathryn Roeder, and Larry Wasserman. Assumption-lean post-integrated inference with surrogate-control outcomes. *Biometrika*, page asag004, 2026.
6. Jin-Hong Du, Larry Wasserman, and Kathryn Roeder. Simultaneous inference for generalized linear models with unmeasured confounders. *Journal of the American Statistical Association*, pages 1–15, 2025.
7. Jin-Hong Du, Zhenghao Zeng, Edward H Kennedy, Larry Wasserman, and Kathryn Roeder. Causal inference for genomic data with multiple heterogeneous outcomes. *Journal of the American Statistical Association*, pages 1–24, 2025.
8. Mariano I Gabitto, Kyle J Travaglini, Victoria M Rachleff, Eitan S Kaplan, Brian Long, Jeanelle Ariza, Yi Ding, Joseph T Mahoney, Nick Dee, Jeff Goldy, et al. Integrated multimodal cell atlas of alzheimer's disease. *Nature Neuroscience*, pages 1–18, 2024.
9. Xin Jin, Sean K Simmons, Amy Guo, Ashwin S Shetty, Michelle Ko, Lan Nguyen, Vahbiz Jokhi, Elise Robinson, Paul Oyler, Nathan Curry, Giulio Deangeli, Simona Lodato, Joshua Z Levin, Aviv Regev, Feng Zhang, and Paola Arlotta. In vivo perturb-seq reveals neuronal and glial abnormalities associated with autism risk genes. *Science*, 370(6520), Nov 2020.
10. Edward H Kennedy, Shreya Kangovi, and Nandita Mitra. Estimating scaled treatment effects with multiple outcomes. *Statistical methods in medical research*, 28(4):1094–1104, 2019.
11. Seunggeun Lee, Wei Sun, Fred A Wright, and Fei Zou. An improved and explicit surrogate variable analysis procedure by coefficient adjustment. *Biometrika*, 104(2):303–316, 2017.
12. Jeffrey T Leek and John D Storey. Capturing heterogeneity in gene expression studies by surrogate variable analysis. *PLoS Genet*, 3(9):1724–35, Sep 2007.
13. Yingxin Lin, Shila Ghazanfar, Kevin YX Wang, Johann A Gagnon-Bartsch, Kitty K Lo, Xianbin Su, Ze-Guang Han, John T Ormerod, Terence P Speed, Pengyi Yang, et al. scmerge leverages factor analysis, stable expression, and pseudoreplication to merge multiple single-cell rna-seq datasets. *Proceedings of the National Academy of Sciences*, 116(20):9775–9784, 2019.
14. Mohammad Lotfollahi, Anna Klimovskaia Susmelj, Carlo De Donno, Leon Hetzel, Yuge Ji, Ignacio L Ibarra, Sanjay R Srivatsan, Mohsen Naghipourfar, Riza M Daza, Beth Martin, et al. Predicting cellular responses to complex perturbations in high-throughput screens. *Molecular systems biology*, 19(6):e11517, 2023.
15. Michael Love, Simon Anders, and Wolfgang Huber. Differential analysis of count data–the deseq2 package. *Genome Biol*, 15(550):10–1186, 2014.
16. Malte D Luecken, Maren Büttner, Kridsadakorn Chaichoompu, Anna Danese, Marta Interlandi, Michaela F Müller, Daniel C Strobl, Luke Zappia, Martin Dugas, Maria Colomé-Tatché, et al. Benchmarking atlas-level data integration in single-cell genomics. *Nature methods*, 19(1):41–50, 2022.
17. Hansruedi Mathys, Zhuyu Peng, Carles A Boix, Matheus B Victor, Noelle Leary, Sudhagar Babu, Ghada Abdelhady, Xueqiao Jiang, Ayesha P Ng, Kimia Ghafari, et al. Single-cell atlas reveals correlates of high cognitive function, dementia, and resilience to alzheimer's disease pathology. *Cell*, 186(20):4365–4385, 2023.
18. Chris McKennan and Dan Nicolae. Accounting for unobserved covariates with varying degrees of estimability in high-dimensional biological data. *Biometrika*, 106(4):823–840, 2019.
19. Chris McKennan and Dan Nicolae. Estimating and accounting for unobserved covariates in high-dimensional correlated data. *Journal of the American Statistical Association*, 117(537):225–236, 2022.
20. Efthymia Papalexi, Eleni P Mimitou, Andrew W Butler, Samantha Foster, Bernadette Bracken, William M Mauck III, Hans-Hermann Wessels, Yuhan Hao, Bertrand Z Yeung, Peter Smibert, et al. Characterizing the molecular regulation of inhibitory immune checkpoints with multimodal single-cell screens. *Nature genetics*, 53(3):322–331, 2021.
21. Yongjin P Park and Manolis Kellis. Cocoa-diff: counterfactual inference for single-cell gene expression analysis. *Genome Biology*, 22(1):1–23, 2021.
22. Davide Risso, John Ngai, Terence P Speed, and Sandrine Dudoit. Normalization of rna-seq data using factor analysis of control genes or samples. *Nat Biotechnol*, 32(9):896–902, Sep 2014.
23. Agus Salim, Ramyar Molania, Jianan Wang, Alysha De Livera, Rachel Thijssen, and Terence P Speed. Ruv-iii-nb: Normalization of single cell rna-seq data. *Nucleic Acids Research*, 50(16):e96–e96, 2022.
24. Bicna Song, Dingyu Liu, Weiwei Dai, Natalie F McMyn, Qingyang Wang, Dapeng Yang, Adam Krejci, Anatoly Vasilyev, Nicole Untermoser, Anke Loregger, et al. Decoding heterogeneous single-cell perturbation responses. *Nature cell biology*, pages 1–12, 2025.
25. Jingshu Wang, Qingyuan Zhao, Trevor Hastie, and Art B Owen. Confounder adjustment in multiple hypothesis testing. *Annals of statistics*, 45(5):1863, 2017.
26. Ethan Weinberger, Chris Lin, and Su-In Lee. Isolating salient variations of interest in single-cell data with contrastivevi. *Nature Methods*, 20(9):1336–1345, 2023.
27. Luke Zappia, Belinda Phipson, and Alicia Oshlack. Splatter: simulation of single-cell RNA sequencing data. *Genome biology*, 18(1):174, 2017.
